# Breastfeeding, introduction of other foods and effects on health: a systematic literature review for the 5th Nordic Nutrition Recommendations

**DOI:** 10.3402/fnr.v57i0.20823

**Published:** 2013-04-12

**Authors:** Agneta Hörnell, Hanna Lagström, Britt Lande, Inga Thorsdottir

**Affiliations:** 1 Department of Food and Nutrition, Umeå, University, Umeå Sweden; 2 Turku Institute for Child and Youth Research, University of Turku, Turku, Finland; 3 Division of Public Health, Norwegian Directorate of Health, Oslo, Norway; 4 Unit for Nutrition Research, School of Health Sciences, University of Iceland and Landspitali National University Hospital, Reykjavik, Iceland

**Keywords:** overweight, infectious disease, atopic disease, celiac disease, diabetes, blood pressure

## Abstract

The present systematic literature review is part of the 5th revision of the Nordic Nutrition Recommendations. The overall aim was to review recent scientific data valid in a Nordic setting on the short- and long-term health effects of breastfeeding (duration of both any and exclusive breastfeeding) and introduction of foods other than breast milk. The initial literature search resulted in 2,011 abstracts; 416 identified as potentially relevant. Full paper review resulted in 60 quality assessed papers (6A, 48B, and 6C). A complementary search found some additional papers. The grade of evidence was classified as convincing, probable, limited-suggestive, and limited-no conclusion. The evidence was convincing of a protective dose/duration effect of breastfeeding against overweight and obesity in childhood and adolescence, overall infections, acute otitis media, and gastrointestinal and respiratory tract infections. The evidence was probable that exclusive breastfeeding for longer than 4 months is associated with slower weight gain during the second half of the first year which could be part of the reason behind the reduced risk of later overweight or obesity. There was also probable evidence that breastfeeding is a protective factor against inflammatory bowel disease, celiac disease, and diabetes (type 1 and 2), provides beneficial effects on IQ and developmental scores of children as well as a small reductive effect on blood pressure and blood cholesterol levels in adulthood. Other associations explored were limited-suggestive or inconclusive. In conclusion, convincing and probable evidence was found for benefits of breastfeeding on several outcomes. The recommendation in NNR2004 about exclusive breastfeeding for 6 months and continued partial breastfeeding thereafter can stand unchanged. The relatively low proportion of infants in the Nordic countries following this recommendation indicates that strategies that protect, support and promote breastfeeding should be enhanced, and should also recognize the benefits for long-term health.

## Background

The World Health Organization (WHO) recommends exclusive breastfeeding during the first six months of life (1–3). From six months onwards, continued breastfeeding combined with complementary foods of good quality in sufficient quantities for 2 years or longer is recommended. Exclusive breastfeeding means that the child only receives breast milk, and if necessary the addition of vitamins, minerals, and medicine. The WHO recommendation applies to all countries and populations regardless of economic status or developmental level.

All the Nordic countries have relatively high breastfeeding rates. After birth, virtually all mothers breastfeed their infants and between 58 and 80% of the infants are still breastfed at 6 months (Table 1). The rate of exclusive breastfeeding is also high in the first months, but decreases quickly to between 23 and 60% of infants exclusively breastfed at 4 months. The majority are introduced to other foods before 6 months of age.

**Table 1:** Reported breastfeeding rates (% exclusive and any breastfeeding) among children born in the Nordic countries (in Denmark little data are available and only proportion of full breastfeeding at 1, 4, and 6 months is available)

	1 w		1 m		2 m		3 m		4 m		5 m		6 m		9 m	12m
							
	Excl	Any	Excl	Any	Excl	Any	Excl	Any	Excl	Any	Excl	Any	Excl	Any	Any	Any
Denmark^1^		95	80						60				12			
Finland^2^			46	87	39	80	34	77	23	68	9	66	0	58	39	34
Iceland^3^	86	98	87	94	80	91	67	86	63	84	35	79	8	74	45	27
Norway^4^			82	95	73	91	63	88	46	85	25	82	9	80	63	46
Sweden^5^	83	97			67	87			51	76			11	63	34	16

1 Children born in 2008 and 2009 in 14 municipalities in Denmark (4).

2 Children born in 2010 Finland. Health and Welfare report 2012 (5).

3 Children born in 2005–06 in Iceland. Nationwide randomized cohort (6) and in 2004–08, Directorate general of Health, Iceland (7).

4 Children born in 2006 in Norway. National dietary surveys (8, 9).

5 Children born in 2010 in Sweden. National statistics 2012 (10).

Breastfeeding has many health benefits for both baby and mother. Breast milk is not only a food source but contains immune-related components and various biologically active substances that contribute to efficient nutrient utilization and gives the child active and passive protection against infections (11). Breastfeeding also provides numerous short- and long-term health benefits. In developing countries, breastfeeding can be the difference between life and death for several reasons including poor hygiene and lack of clean water. The impact of breastfeeding on mortality rates among healthy full-term infants in developed countries is unclear due to a scarcity of studies focused on this matter. However, there is clear evidence that the child's morbidity is affected, notably with the increased risk of gastroenteritis and acute otitis media. For other diseases the level of evidence is weaker and variable. However, in a Nordic setting infant formula is a safe option if breastfeeding is not possible.

In 2010, the Nordic Council of Ministers launched a project aimed at reviewing the scientific basis of the NNR issued in 2004 (12) and as necessary, to update the guidelines for the 5th edition. The NNR5 project is mainly focused on revising the areas in which new scientific knowledge, with special relevance to the Nordic setting, has emerged since the 4th edition. A number of systematic literature reviews (SLRs) will form the basis for the update.

The present SLR is focused on breastfeeding and introduction of solid food and the association with several different health outcomes.

## Aims

The overall aim was to review recent scientific data valid in a Nordic setting on the short- and long-term health effects of breastfeeding (duration of both any and exclusive breastfeeding) and introduction of foods other than breast milk in order to assess the validity of the current Nordic recommendations. A second aim was to provide a background for the planned update on the chapter on breastfeeding.

### Research/key questions

1. What are the associations between duration of exclusive breastfeeding/any breastfeeding and growth in infancy, or overweight and obesity in later life?
2. What are the associations between introduction of foods other than breast milk and growth in infancy, or overweight and obesity in later life?
3. What is the association between duration of exclusive breastfeeding/any breastfeeding and atopic disease, asthma, or allergy?
4. What is the association between introduction of foods other than breast milk and atopic disease, asthma, or allergy?
5. What are the associations between duration of exclusive breastfeeding/any breastfeeding and health and disease outcomes in infancy and later in life, such as: infectious diseases (otitis media, gastrointestinal infections, and respiratory tract infections), cognitive and neurological development, cardiovascular disease (CVD), cancer, diabetes, blood pressure, glucose tolerance, and insulin resistance?

Limits: published since January 2000, human subjects. See below for inclusion and exclusion criteria and Appendix 1 for search terms.

Originally we also intended to include the effects of breastfeeding on maternal health but due to time constraints it was decided to postpone this part.

## Methods

### Search terms

Search terms were defined during spring 2011, in collaboration with Sveinn Olafsson, librarian at Landspitali-University Hospital, Reykjavik, Iceland. The search terms are presented in Appendix 1.

### Inclusion and exclusion criteria

The group focused on breastfeeding and the introduction of solid foods to healthy, full-term children by healthy mothers. Inclusion criteria in the abstract screening process were the following: English or Nordic language, study population relevant to the Nordic countries. Studies were excluded if the exposure was pro- and/or prebiotics, special formulas e.g. containing added long-chain poly-unsaturated fatty acids (LC-PUFA), supplements to mother or infants, contamination of breast milk e.g. lead and mercury, if mother or child was sick at start or at increased risk for disease, or if the outcome was other than those stated in the research questions. Cross-sectional studies only describing breastfeeding status without relevant outcomes of interest for this review were also excluded.

Dietary studies are often methodologically problematic and studies on breastfeeding are no exception. It has been shown that retrospective studies where parents are asked to recall infant feeding data hava low reliability (13). Studies with a recall periods longer than 3 years were therefore excluded.

Due to the high rates of breastfeeding in the Nordic countries, studies where breastfeeding was only defined as never or ever breastfed, where the definition *ever* could include everything between being put to the breast at the maternity ward to several years of breastfeeding, were deemed inapplicable for the Nordic recommendations and these studies were excluded. However, the included SLRs and meta-analyses (MAs) could include studies with recall periods longer than 3 years or where breastfeeding was only defined as never or ever breastfed.

Papers that were incorporated into an included SLR/MA or published before the search years of an included SLR/MA with the same outcome were excluded before the full paper screening.

### Search results

**Fig. 1:**
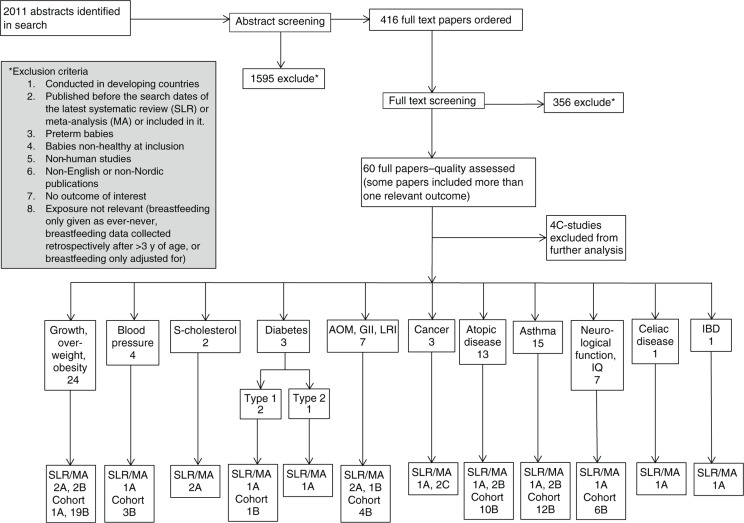
Overview search results of SLR on breastfeeding/introduction of other foods and health outcomes.

The final search was run in June 2011, including all the relevant population groups and clinical outcomes, resulting in 2,011 abstracts (Fig. 1). An additional 1,026 abstracts were classified as overviews or reviews but did not include the description ‘systematic review’ or ‘meta-analysis’ and we therefore decided (together with our librarian) that these most likely were ‘overviews’ of the area rather than proper systematic reviews, and these were therefore not included. Abstract screening was conducted in July and August 2011 according to the guide for conducting SLRs for the 5th edition of the NNR (14).

In total 416 full papers were ordered, including 40 SLR/MA. Of these 416 papers, 214 were immediately excluded for the following reasons: 74 papers were incorporated into an included SLR/MA, 70 papers were published before the search years of an included SLR/MA with the same exposure and outcome, 49 papers on maternal health, eight commentaries, opinions or letters to the editors, six overviews, six papers with a population not applicable to Nordic countries today, and one workshop. Full paper screening was conducted for the remaining 202 publications September–December 2011, where 142 further papers were excluded. Reasons for exclusion are provided in Appendix 2. Finally, 60 papers were selected for quality assessment; 13 SLR/MA, 41 prospective cohort studies, and six originating from the Promotion of Breastfeeding Intervention Trial (PROBIT) study.

The PROBIT-study is a cluster-randomized intervention trial in Belarus where geographical sites were randomized to interventional breastfeeding promotion or controls. The experimental intervention led to a large increase in exclusive breast feeding at 3 months (44.3% v 6.4%; p<0.001) and a significantly higher prevalence of any breastfeeding at all ages up to and including 12 months. Power calculation for the study was only performed for the outcome gastroenteritis. Results from the study are presented in several papers where either differences between the intervention and control areas, or between exclusive breastfeeding 3 months (EBF3) or 6 months (EBF6), or effects of duration of any breastfeeding in months in relation to different outcomes are described.

Detailed information about all graded SLR/MAs and prospective cohort studies are found in Appendix 3 and 4, respectively. Papers originating from the PROBIT-study are presented in Appendix 4 together with the prospective cohort studies.

In addition to these original papers, we included 13 reports originating from various organizations and associations; American Academy of Pediatrics (AAP), Committee on Toxicity of Chemicals in Food Consumer Products and the Environment (COT), European Food Safety Authority Panel on Dietetic Products, Nutrition and Allergies (EFSA-NDA), European Society for Pediatric Gastroenterology, Hepatology, and Nutrition (ESPGHAN), EU FP6 project ‘The Prevalence, Cost and Basis of Food Allergy Across Europe’ (EuroPrevall), Scientific Advisory Committee on Nutrition (SACN), Swedish Pediatric Society, and the WHO. Details about the included reports are given in Appendix 5.

A complementary search was conducted at the end of January 2012 covering the period between the first search until the end of December 2011. The abstracts were similarly evaluated for full paper reading. Included complementary papers were quality assessed and used to evaluate the conclusion of the SLR, as supporting or not.

### Quality assessment, grading and reporting of evidence

The 60 included papers were quality assessed using the Quality Assessment Tools (QAT) received from the NNR5 secretariat (14), which included a modified assessment of multiple systematic reviews (AMSTAR) for assessment of SLRs/MAs. These contained a number of questions regarding several methodological aspects of the studies including study design, population characteristics, exposure and outcome measures, dietary assessment, and confounders. The PROBIT-study papers were assessed as prospective cohort studies.

The quality assessment resulted in the following grading; SLR/MA: 5A, 5B and 3C; prospective cohort studies (including PROBIT): 1A, 43B, and 3C.

The findings for each separate outcome are presented in Supplementary Tables 1–11. Only studies graded A and B are included in these tables, except for Supplementary Table 6 which also includes two SLRs graded C as the number of studies with cancer as the main outcome was low.

All steps in the process of selecting and grading papers, i.e. abstract screening, paper screening, and quality assessment, was performed as described in the guide for conducting SLRs. This meant that each abstract/paper was evaluated by two experts. The experts first made an individual appraisal which then was discussed and a joint conclusion was made.

The grade of evidence was classified as convincing (grade 1), probable (grade 2), limited-suggestive (grade 3), and limited-no conclusion (grade 4) depending on the number and quality of supporting, non-supporting, and contradicting studies.

## Results

Nine of the 13 reports originating from various organizations and associations and published during recent years, give a more general conclusion on the relationship between breastfeeding and health outcomes and these conclusions are summarized here in the paragraphs below. Conclusions on specific diseases are given in the relevant chapters.

AAP (15, 16), EFSA (17), ESPGHAN (18, 19), SACN (20), and WHO (21), all conclude that breastfeeding and the use of human milk confer unique nutritional and non-nutritional benefits to the infant and the mother and, in turn, optimize infant, child, and adult health as well as child growth and development. These organizations reaffirm their recommendations of exclusive breastfeeding for approximately 6 months, followed by continued breastfeeding as complementary foods are introduced, with continuation of breastfeeding up to 1 year or longer [WHO (21) – 2 years or longer] as mutually desired by mother and infant.

EFSA (17) and ESPGHAN (19) have looked into the risks of introducing complementary feeding before 6 months of age. EFSA (17) states that the introduction of complementary food in the diet of healthy term infants in the EU, between the ages of 4 and 6 months, is safe and does not pose a risk for adverse health effects. ESPGHAN Committee on Nutrition adds that complementary feeding should not be introduced before 17 weeks and not later than 26 weeks (19), and that partial breastfeeding as well as breastfeeding for shorter periods of time are also beneficial (18).

All these organizations agree that strategies that protect, support and promote exclusive breastfeeding for the first 6 months of an infant's life should be encouraged and also recognize the benefits for long-term health.

### General growth and overweight/obesity

Supplementary Table 1 shows a summary of studies with the main outcome growth and overweight/obesity (details are provided in Appendix 3–5). In total, 23 papers were chosen in the systematic review process to be evaluated for the evidence of an association between breastfeeding and weight development, i.e. later overweight or obesity. Of those, 19 were prospective cohorts (including birth cohorts) and four were SLR/MA. Three relevant reports are also described.

#### SLR/meta-analysis

Ip et al. (22) conducted a SLR graded A, including 3 SLR or meta-analysis (a total of 61 studies: 35 observational, 18 cohort, seven cross-sectional, one case-control), on overall breastfeeding (defined as ever vs. never, in one study breastfeeding >2 months vs. never) and overweight. Ip et al. concluded that a history of breastfeeding was associated with reduced risk of obesity in childhood and/or adult life, but state that the observed association could reflect selective reporting and/or publication bias. Pooled adjusted odds ratio (OR) was 0.76 (95% confidence interval [CI]: 0.67, 0.86), 0.93 (95% CI: 0.88, 0.99) and 0.96 (95% CI: 0.94, 0.98) in three included studies for those breastfed compared with never breastfed.

The SLR by Kramer and Kakuma (2), graded A, included only controlled clinical trials and observational studies. The aim was to examine whether or not exclusive breastfeeding for 6 months had an impact on growth (and other outcomes). They concluded that infants breastfed exclusively for 6 or more months had no observable deficits in growth (up to 12 months of age).

Monasta et al. (23) studied the evidence for early-life determinants of obesity (to 5 years of age) in a review of systematic reviews. They reported that no/short breastfeeding was one of five factors associated with overweight and obesity in childhood and/or adult life. This was supported by better-quality reviews. They included 22 reviews of which eleven were of moderate quality and eleven of low quality. Articles published after the reviews were used to confirm results. They concluded that breastfeeding may be a protective factor against overweight and obesity. For odds ratios, see Supplementary Table 1.

An SLR by Moorcroft et al. (24) did not find any clear association between the age of introduction of solid foods and obesity in infancy and childhood. In total, 24 papers were included in the SLR (one RCT, re-analysis of data from two RCTs, 20 cohort studies and one case-control study). Eight papers measured outcomes up to 1 year of age; one found a positive association between early introduction of solids and weight at 6 months, one found a positive association at 6 months but not at 1 year, three found a positive association at 1 year and three found no association between age at introduction and later weight. Nineteen papers measured outcomes after 1 year and up to 18 years of age (one also mentions 42 years of age). Of these, four found a positive association between early introduction of solids and later weight, while the remaining 15 found no association. The reverse was never seen.

#### Prospective cohort studies

The WHO working group on the Growth Reference Protocol and the WHO Task Force on Methods for the Natural Regulation of Fertility (25) aimed to study the impact of timing, frequency and type of complementary foods in a prospective study. The study was graded B. Five to seven hundred mothers and infants were recruited within the first week after birth in all seven countries (only two were Western countries) to be prospectively followed for 8 months. Study site was adjusted for in the analysis, but no major differences in growth related to breastfeeding were seen. Analysis performed on data from 1,252 infants showed significantly slower growth (both length and weight) during the first 4 months for those infants given complementary food before the age of 4 months compared to those introduced to complementary foods between 4 and 6 months of age. Infants who were given complementary foods after reaching 6 months of age had significantly slower length velocity from 4 to 8 months of age when compared to those given complementary foods between 4 and 6 months of age. Concerning analysis of type and frequency of complementary foods and breastfeeding, from 4 to 6 months of age the frequency of receiving solid foods was positively associated with length velocity and the frequency of breastfeeding negatively with weight velocity. Based on the modest but significant effects found, the authors concluded that the results did not provide adequate evidence of benefit or risk related to timing of complementary foods and growth nor to differential types and frequencies of complementary foods between 4 and 6 months of age in healthy infants living in environments without major economic restraints and low rates of illness.

Aarts et al. (26) published a sub-study of the WHO multicentre study (25) to test exclusive vs. non-exclusive breastfeeding in Sweden. The study was graded B. The conclusion of this study was that truly exclusively breastfed children for the first 6 months grew similar to those not exclusively breastfed from the age of 3–4 months (however the latter group had also a high rate of breastfeeding).

Chivers et al. (27) reported an analysis from the Raine pregnancy cohort from Western Australia which showed a significantly higher frequency of overweight and obesity when breastfeeding was stopped before 4 months of age vs. continued breastfeeding for a longer period of time. Similar results were found when analyzing the introduction of other milk before the age of 4 months vs. after. Body mass index (BMI, kg/m^2^) was measured at the age of 1, 2, 3, 6, 8, 10, and 14 years and found to be higher over time for those breastfed shorter than 4 months or given other milk before that age. The authors concluded that early infant feeding was important for timing of adiposity rebound, and that early infant feeding had effects on BMI up to the age of 14 years. Their findings support the importance of exclusive breastfeeding for duration longer than 4 months.

Cole et al. (28) used a sub-sample (n=120) of the Cambridge Infant Growth Study to construct new median weight curves for breastfed infants and compare it with the British 1990 reference curve. The infants were fed breast milk (with no formula) for at least 24 weeks, with solids introduced at a mean age of 15 weeks. They concluded that the British 1990 reference curves reflected the growth of long-term breastfed infants only imperfectly, with mean weight for these infants falling by 0.5 standard deviation scores (SDS) from 2 to 12 months.

De Hoog et al. (29) studied ethnic differences in growth (SDS weight, length, weight-for-length) during first 6 months among infants born in the Netherlands. Infant feeding was defined as duration of breastfeeding: (none, 1 month, 1–3 months, 4–6 months, and >6 months); age at the introduction of formula feeding (none, 1 month, 1–3 months, 4–6 months, and >6 months); age at the introduction of complementary food (<4 months, 4 months, 5 months, and >5 months). The growth rate was higher in almost all ethnic minorities, with β between 0.07 and 0.41 for weight and between 0.12 and 0.42 for length, compared with ethnic Dutch infants. In general, exclusive breastfeeding for 4 months was associated with slower growth for all three growth measures compared with those not exclusively breastfed. Feeding factors explained a small degree of the higher weight and length gain in infants of African descent, but not a higher SDS weight-for-length in the Moroccan population.

De Kroon et al. (30) studied the association between duration of exclusive breastfeeding on BMI and body fat at 18–28 years of age (estimated by validated questionnaire) in the Netherlands. Infant feeding practices were recorded during repeated health visits in infancy, but definition of exclusive breastfeeding only included not giving infant formula and other foods were not considered. Significant inverse dose-response of breastfeeding duration was found for BMI (β−0.13, SE 0.06), waist circumference (β−0.39, SE 0.18) and waist-hip-ratio (β−0.004, SE 0.001) after correction for age, gender, and confounders. A relation between exclusive breastfeeding and more positive dietary behavior was also reported.

Another Dutch study, Durmus et al. (31) found no association between the duration of any or exclusive breastfeeding with growth rates between 0 and 3 months. Shorter breastfeeding was associated with increased gain in age- and sex adjusted SDS for length, weight, and BMI between 3 and 6 months (*p* for trend <0.05). Similar tendencies were seen for breastfeeding exclusivity. Breastfeeding duration and exclusivity were not consistently associated with risk of overweight and obesity at 1, 2, and 3 years of age.

Gubbels et al. (32) compared weight gain in the first year, and BMI and overweight up to age 4 years between breastfed and formula-fed infants in the KOALA Birth Cohort Study from the Netherlands. Breastfeeding duration was studied up to 12 months of age. Each additional month of breastfeeding was associated with less weight gain in the first year (β-37.6, *p* <0.001), a lower BMI z-score at age 1 (β−0.02, p<0.01), and lower odds of being overweight at 1 year of age (OR=0.96, *p* <0.05). No significant associations between breastfeeding and BMI or overweight were found at ages above 1 year.

In a prospective cohort, Huh et al. (33) studied introduction of solid foods at <4 months of age, 4–5 months of age or ≥6 months separately among infants breastfed more than 4 months, never breastfed, or stopped breastfeeding before 4 months of age (formula feds) and possible obesity at 3 years of age. Among breastfed infants timing of solid food was not associated with obesity at 3 years of age (OR 1.1 [95% CI: 0.3, 4.4]). Among formula-fed infants (or weaned before 4 months of age), introduction of solid foods before 4 months of age was associated with obesity at 3 years of age (OR 6.3 [95% CI: 2.3, 6.9]), but it was not explained by rapid early growth.

Kalies et al. (34) found in a prospective cohort from Germany that those who were exclusively breastfed <6 months had a greater risk of elevated weight gain at 2 years of age compared with children breastfed for 6 months and more (OR 1.65 [95% CI: 1.17, 2.30]). However, they do not include solids in their definition of exclusive breastfeeding. Duration of exclusive breastfeeding was inversely associated with the risk of elevated weight gain in a strongly duration-dependent way. Infants exclusively breastfed ≤1 months had twice as often elevated weight gain (OR 1.99 [95% CI: 1.34, 2.97]) compared to infants breastfed ≥6 months. Duration of exclusive breastfeeding was defined as the number of months breastfed without concomitant feeding of infant's formula and classified a priori as <6 months or ≥6 months, and for dose-response-analysis in the categories: 0–1 months, 2–3 months, 4–5 months, ≥6 months.

In another prospective cohort study, Kitsantas and Gaffney (35) found that preschoolers with normal BMI had been breastfed longer than their overweight/obese peers (mean [SD]: 1.9 [2.7] months vs. 1.7 [2.5] months). However, while shorter duration of breastfeeding did emerge as a risk, it was not a significant predictor in the logistic regression analysis.

Three papers from the PROBIT-study focus on various indicators of growth. In a paper from 2007, including 13,889 of 17,046 total participants, Kramer et al. (36) found no significant intervention effects at 6.5 years on adiposity, stature, height, waist or hip circumference, triceps or subscapular skinfold thickness. They conclude that previously reported beneficial effects on these outcomes may be the result of uncontrolled confounding and selection bias. However, in a later paper from 2009 (37) which included 2,951 of 3,483 total participants followed during the first year, they report that BMI, triceps skinfold thickness, and hip circumference at the age of 6.5 years were higher among children exclusively breastfed for 6 months compared with those exclusively breastfed for 3 months. Another study from Kramer et al. (38) concluded that smaller size (especially weight for age) was strongly associated with increased risks of subsequent weaning and discontinuing exclusive breastfeeding (adjusted OR 1.2–1.6), especially between 2 and 6 months of age, even after adjustment for potential confounding factors and clustered measurement.

Oddy et al. (39) reported that infants overweight at 52 weeks were more likely to have been given formula feeds at an earlier age than normal weight infants. Infants who had been fully breastfed for at least 4 weeks were lighter (9,731 g vs. 10,138 g; *p*=0.041) and shorter (73.7 cm vs. 75.6 cm; *p*=0.001) than infants who had received infant formula by 4 weeks. These results were stronger for boys and for babies less than 3,500 g at birth.

Rebhan et al. (40) described the effects of the application of the WHO recommendation of exclusive breastfeeding to 6 months of age on infants’ growth up to the age of 9 months. The mothers were retrospectively questioned about breastfeeding when their children were 9 months old. Those breastfed shorter than 4 months showed lower weight-for-length z-scores in the first days of life and higher z-scores in months 6 and 7 than those exclusively breastfed for longer than 4 months. No significant difference in growth was found between those exclusively breastfed for 4–6 months vs. 6 months or more.

Rzehak et al. (41) evaluated the effect of exclusive breastfeeding for 4 months on weight, length, and BMI by regular measurements up to the age of 6 years. Those exclusively breastfed for 4 months gained less weight, but grew equally in length in the first 12 months of life vs. those children on mixed feeding or only formula. Length velocities were not different between the groups. Over time, a slightly lower risk (difference <2%) of being overweight was estimated for infants exclusively breastfed for 4 months. The protective effect of breastfeeding on becoming overweight was related to its weight-velocity-modifying-effect in early infancy. The drawback of this study is that it does not take different duration of breastfeeding beyond 4 months into account.

Scholtens et al. (42) found that children breastfed for more than16 weeks had a lower BMI at 1 year of age than non-breastfed infants (after adjustment for confounders; β−0.22, 95% CI: −0.39, −0.06). The association between breastfeeding and BMI between 1 and 7 years of age was negligible, while a high BMI at 1 year of age was strongly associated with a high BMI between 1 and 7 years of age in the same model. These findings suggest that the lower BMI and lower risk of overweight among breastfed children later in life are already achieved at 1 year of age. In a later follow-up of the same children, Scholtens et al. (43) reported that breastfeeding for over 16 weeks was significantly associated with a lower overweight risk at 8 years [OR 0.67 (95% CI: 0.47, 0.97)], and the association hardly changed after adjustment for diet [OR 0.71 (95% CI: 0.49, 1.03)].

Van Rossem et al. (44) reported that at the age of 3 years, adjusted BMI z-score of fully breastfed infants at 6 months was 0.17 (95% CI: −0.43, 0.09) units lower than those never breastfed. After additional adjustment for infant weight change, the estimate for BMI z-score was attenuated (−0.03, 95% CI: −0.27, 0.20). Similar results were seen with the sum of subscapular (SS) and triceps (TR) skinfold thicknesses. For each month a child was breastfed until the age of 6 months, the decrement in BMI z-score was 0.04 units (95% CI: −0.07, −0.01) and the decrement in SS+TR was 0.19 mm (95% CI: −0.31, −0.07) and the odds of being obese was reduced by 8% (95% CI: −2%, −18%).

#### Reports

SACN (20) conclude that infants who are breastfed are less likely to be obese in later life.

A report from WHO (21) states that the risk for overweight/obesity in childhood and adolescence was 20% (1–9 year) to 30% (9–19 year) lower among breastfed subjects compared with non-breastfed. Difference for >19 years was not significant and the pooled OR was (95% CI) 0.79 (0.71–0.87), 0.69 (0.60–0.80), and 0.88 (0.74–1.04), respectively.

WHO undertook the Multicentre Growth Reference Study between 1997 and 2003 to generate new curves for assessing the growth and development of children the world over using breastfed children as the norm (45). Primary growth data and related information were gathered from 8,440 healthy breastfed infants and young children from widely diverse ethnic backgrounds and cultural settings (i.e. Brazil, Ghana, India, Norway, Oman, and USA). The study resulted in new growth charts showing slower growth of the breastfed infants from about 2–3 months of age compared to previous international growth charts of infants given formula and other food.

#### Conclusion

The majority of studies included in the present SLR reported duration of breastfeeding without distinguishing between the time children were exclusively and partially breastfed. In addition age of outcome measurements varied: during the first year of life (seven studies), toddlers/preschool age (10 studies) and from school-age up to adulthood (six studies, including one with varied ages).

#### Exclusive or any breastfeeding and growth in infancy

Seven studies reported associations of exclusive breastfeeding, either as a continuous variable or for 3, 4, or 6 months, with growth. Four studies found no difference in growth between those exclusively breastfed for 4 months or 6 months (2, 25, 26, 40). Rebhan et al. (40) also found that those exclusively breastfed less than 4 months showed higher weight-for-length z-scores at 6–7 months compared with those exclusively breastfed for 4 months or longer.

Two studies found that infants exclusively breastfed for 4 months showed slower growth during the first 6 months (29) or 3–6 months (31) compared with those with shorter breastfeeding. Similarly, Rzehak et al. (41) found that infants exclusively breastfed for 4 months gained less weight, but grew equally in length in the first 12 months of life vs. children on mixed feeding or only formula.

Kramer et al. (38) found that smaller size (especially weight for age) was strongly associated with increased risks of subsequent weaning and discontinuing exclusive breastfeeding (adjusted OR 1.2 –1.6), especially between 2 and 6 months of age.

#### Exclusive breastfeeding and risk of overweight/obesity

Three prospective cohort studies found a lower risk of overweight or obesity with longer duration of exclusive breastfeeding. Oddy et al. (39) found that infants who had been fully breastfed for at least 4 weeks were lighter and shorter at 52 weeks than infants who had received infant formula by 4 weeks. Rzehak et al. (41) found a slightly lower risk (difference <2%) of being overweight at 6 years of age for those exclusively breastfed for 4 months compared with children on mixed feeding or only formula. Additionally, Huh et al. (33) found that earlier introduction of solids among children fed formula before 4 months of age was associated with obesity at 3 years of age, but among breastfed infants the timing of introduction of solid foods was not associated with obesity.

In contrast, Durmus et al. (31) found no consistent association between breastfeeding duration and exclusivity with risk of overweight and obesity at age 1, 2, and 3 years, and Kramer et al. (37), reported that BMI, triceps skinfold thickness, and hip circumference at 6.5 years of age were higher among children exclusively breastfed for 6 months compared with those exclusively breastfed for 3 months.

#### Duration of breastfeeding and risk of overweight/obesity

One SLR and eight prospective cohort studies show lower risks of overweight and/or obesity with longer breastfeeding duration. Chivers et al. (27) found a higher BMI over time up to 14 years of age for those breastfed shorter than 4 months or given other milks before this age. De Kroon et al. (30) found a significant inverse dose-response of breastfeeding duration for BMI and body fat at 18–28 years of age. Gubbels et al. (32), studied breastfeeding duration up to 12 months of age and found that each additional month of breastfeeding was associated with less weight gain, a lower BMI z-score at age 1 year, and lower odds of being overweight in the first year of life, but not at ages above 1 year. Kalies et al. (34) found that those who were breastfed <6 months had a greater risk of elevated weight gain at 2 years of age compared with those exclusively breastfed for 6 months or longer. They also found that duration of exclusive breastfeeding^1^ was inversely associated with the risk of elevated weight gain in a strongly duration-dependent way. Monasta et al. (23), concluded that breastfeeding may be a protective factor against overweight and obesity. Kitsantas and Gaffney (35) found that preschoolers with normal BMI had been breastfed longer than their overweight/obese peers. Scholtens et al. found that compared with non-breastfed children, those breastfed for more than 16 weeks had a lower BMI at 1 and 8 years of age (43), but not at age 7 (42). Van Rossem et al. (44) found that for each month a child was breastfed until the age of 6 months, the odds of being obese at 3 years was reduced by 8% (95% CI: −2%, −18%).

In contrast, Kramer et al. (36) found no significant differences between the intervention area and the control area at 6.5 years on growth indices and suggest that previously reported beneficial effects on these outcomes may be the result of uncontrolled confounding and selection bias.

An SLR by Moorcroft et al. (24), studied age of introduction of solid foods and obesity and found no clear associations.

Based on the above, we conclude that growth in infancy (length and height) varied only a little between those exclusively breastfed for 4 months or 6 months. Nonetheless, there is probable evidence (grade 2) that exclusive breastfeeding for longer than 4 months is associated with slower weight gain during later infancy compared with those exclusively breastfed for less than 4 months of age. This is also supported by the new WHO child growth standards (45), when compared to the old international growth reference, which was mainly based on non-breastfed infants.

The evidence is convincing (grade 1) that longer duration of exclusive breastfeeding or any breastfeeding is associated with a protective effect against overweight and obesity in childhood and adolescence. This is also supported by SACN (20) and WHO (21). To further the conclusion, three studies show a dose-response relationship with longer duration giving more protection (30, 32, 34).

With regard to the association with overweight/obesity in adulthood, due to a scarcity of strong studies, we judge the evidence as limited-suggestive (grade 3) for a protective effect of breastfeeding.

### Blood pressure

Supplementary Table 2 shows studies with outcome blood pressure during childhood and/or later in life (details are provided in Appendix 3–5). In total four papers were chosen in the systematic review process to be evaluated for the evidence of an association between breastfeeding and blood pressure. Three of those were prospective cohorts (including birth cohort), all graded B, mainly due to a lack of power calculations, and one was a meta-analysis, graded A. There are also two reports on the associations on breastfeeding and blood pressure (20, 21).

#### SLR/meta-analysis

Ip et al. (22) performed a SLR (A-graded) on two MAs graded B, one of which included the age group 1–60 years and the other 1–71 years. In total the two MAs included 24 studies of various design (observations within randomized controlled trials [RCTs], prospective cohorts, retrospective cohort, and cross-sectional studies), 13 of the studies were included in both MAs. Ip et al. did not combine the two meta-analysis but concluded that systolic blood pressure in age groups 1–60 years and 1–71 years were lower by −1.4 (95% CI: −2.2, −0.6) and −1.10 (95% CI: −1.8, −0.4), respectively, in the group of breastfed compared with formula fed. The same trends were seen in diastolic blood pressure, which was lower among breastfed (age group 1–60 years −0.5 [95% CI: −0.9, −0.04] and 1–71 years −0.36 [95% CI: −0.79, 0.08]). Although both MAs had moderate methodological quality and reported similar findings, the authors had different appraisals of the public health importance of the small reduction in systolic blood pressure. Ip et al. therefore concluded that a history of breastfeeding during infancy has small reductive effect to adult blood pressure, but the clinical or public health implication of the finding is unclear.

#### Prospective cohort studies

De Jonge (46) compared left cardiac structures and blood pressure at 2 years of age among three groups of children; never breastfed, partially breastfed, and exclusively breastfed for ≥4 months. They found no differences in cardiac structures and blood pressure at age 2 years between breastfed and non-breastfed children. Duration and exclusivity of breastfeeding was not consistently associated with outcomes.

Two papers from the PROBIT-study (36, 37), focus on blood pressure at the age of 6.5 years and found no significant differences in blood pressure neither between the intervention and the control areas (36) nor between those exclusively breastfed for 3 months vs. 6 months (37).

#### Reports

It is stated in a WHO report from 2007 (21) that subjects who were breastfed experienced lower mean blood pressure in later life. The difference was statistically significant, but the magnitude was relatively modest (decreased by slightly more than 1 mmHg).

Similarly, in a report from 2011, SACN (20) conclude that infants who are breastfed tend to have slightly lower blood pressure although there is inconsistent evidence that breastfeeding influences subsequent cardiovascular mortality.

#### Conclusion

The SLR by Ip et al. (22) (including 24 studies) found an association between breastfeeding and lower systolic blood pressure. The three prospective studies found no association between feeding history and blood pressure in childhood. However, the two reports below found the evidence to be in line with the SLR by Ip et al. (22); that is, breastfeeding has a small but significant reductive effect on blood pressure. In the present SLR, we therefore judge that there is (at least) probable evidence (grade 2) for this association.

### Serum cholesterol

Supplementary Table 3 shows two SLRs, both graded A, chosen in the systematic review process to be evaluated for the evidence of an association between breastfeeding and later cholesterol levels (details are provided in Appendix 3). There are also two reports on the associations on breastfeeding and serum cholesterol in adulthood (20, 21).

#### SLR/meta-analysis

Ip et al. (22) reviewed if breastfeeding (ever vs. never) is associated with cholesterol levels in infancy (<1 year), childhood/adolescence (1–16 years) and/or adulthood (17–65 years). They looked at one meta-analysis (graded C) including 27 cohort and 13 cross-sectional studies; infants (26 studies), children/adolescents (17 studies), and adults (nine studies). In 25 of 26 observations, infants who were breastfed had higher mean total cholesterol levels compared with infants who were formula-fed. In 16 of 17 observations in children or adolescents, the mean total cholesterol levels for those who were breastfed in their infancy were similar to those who were formula-fed. In seven of nine observations on adults, lower mean total cholesterol levels were reported for those breastfed in their infancy compared with those who were formula-fed. However, Ip et al. state that no conclusions could be drawn about the evidence based on this meta-analysis since it was graded C (due to data based on individuals with a wide age range, gender, and other confounders were not explicitly analyzed, no detailed information (e.g. fasting or not fasting) on the collection of specimen for cholesterol testing included).

Owen et al. (47) performed a SLR and examined whether breastfeeding is associated with lower total cholesterol concentrations in adulthood. They explored data from 17 studies (10 prospective cohorts, four cross-sectional, two historical cohorts, one retrospective cohort) and found that ever breastfed had lower cholesterol levels than those never breastfed (−0.04 mmol/L [95% CI: −0.08, 0.00], *p*=0.037). The difference in cholesterol between infant feeding groups was larger (p=0.005) and more consistent in seven studies that analyzed ‘exclusive’ feeding patterns (−0.15 mmol/L [95% CI: −0.23, −0.06]) than in ten studies that analyzed non-exclusive feeding patterns (−0.01 mmol/L [95% CI: −0.06, 0.03]).

#### Reports

A WHO report from 2007 (21) states that subjects who had been breastfed experienced lower total cholesterol compared with non-breastfed. The difference was statistically significant, but the magnitude was relatively modest (mean difference: −0.18; 95% CI: −0.30, −0.06 mmol/L).

Similarly, in a report from 2011, SACN (20) concluded that infants who are breastfed tend to have slightly lower total serum cholesterol concentrations in adult life although there is inconsistent evidence that breastfeeding influences subsequent cardiovascular mortality.

#### Complementary search

Bekkers et al. (48), graded B, investigated the influence of breastfeeding and other perinatal risk factors on total and high density lipoprotein (HDL) cholesterol concentrations (non-fasting state) in 8-year-old children in the PIAMA birth cohort study in the Netherlands. Total breastfeeding duration was assessed in infancy and categorized as no breastfeeding, breastfeeding for 1–16 weeks, or breastfeeding for 16 weeks or longer. No significant associations were found between breastfeeding and total cholesterol or HDL concentrations.

#### Conclusion

Both SLRs included in the present SLR (22, 47) showed that breastfeeding might be beneficial and have lowering effects on blood cholesterol concentrations in adulthood, although Ip et al. (22) concluded that the quality of the meta-analysis was too poor to draw conclusions from. However, two reports, WHO 2007 (21) and SACN 2011 (20) found a small but consistent relation between breastfeeding and lower blood cholesterol later in life. In the present SLR, we judge it to be (at least) probable evidence (grade 2) for a small reduction on blood cholesterol in adulthood from breastfeeding but there is less evidence for an association between breastfeeding and blood cholesterol in childhood. This conclusion is supported by a recent paper of Bekkers et al. (48) on cholesterol in childhood, found by the complementary search to the present SLR.-

### Diabetes mellitus (T1DM and T2DM)

Supplementary Table 4 shows studies with outcome diabetes type 1 (T1DM) and type 2 (T2DM) (details are provided in Appendix 3–5). In total, two papers were chosen in the systematic review process to be evaluated for the evidence of an association between breastfeeding and diabetes. Of those, one was a prospective cohort (49), and the other was a meta-analysis (22) including both T1DM and T2DM as outcomes. There was also one report on the associations on breastfeeding and T1DM (20) and two on T2DM (20, 21).

#### SLR/meta-analysis

Ip et al. (22) summarize two meta-analysis (performed in 1994 and 1996, respectively) and six later studies on the association between breastfeeding and the risk of T1DM. The two meta-analysis were of fair quality and included a total of 17 case-control studies reported OR 1.23 (95% CI: 1.12, 1.35) and 1.43 (95% CI: 1.15, 1.77), respectively, for the risk of T1DM if breastfeeding duration was less than 3 months compared to breastfeeding for more than 3 months. Five of the six later studies show similar results; however, these were retrospective case-control studies. Comparison of ORs between studies with long-term recall of breastfeeding data and those more recent showed significant differences in T1DM risk only with long-term retrospective data.

Ip et al. (22) also looked at T2DM in a pooled analysis of seven studies (three historical cohort, two cross-sectional, one prospective cohort, and one case-control studies). Pooled adjusted OR was 0.61 (95% CI: 0.44, 0.85) for those breastfed compared with formula fed. However, only three of the seven studies had information about important confounders, and although these three studies concluded that adjustment did not alter crude estimate, the authors do not feel confident that all potential confounders have been ruled out.

#### Prospective cohort studies

Couper et al. (49) followed prospectively an Australian birth cohort of 548 infants (Baby Diab study, Melbourne) until 6 years of age to investigate the relationship between early growth and infant feeding and the risk of islet cell autoimmunity. They analyzed breastfeeding, exclusive and total as none, duration 0–3 months, and duration >3months. They also evaluated the time of introducing cow's milk, gluten-, and non-gluten food as well as breastfeeding at introduction of cereals and cow's milk. Unfortunately there was a significant amount of missing data in the diet records restricting the power of the analysis and as noted by the authors, an effect could have been missed. The study showed no effect of breastfeeding or other diet variables, but being above average in weight in early life, especially the first 2 years, increase the risk of islet cell autoimmunity in children with a first degree relative with T1DM. The authors point out the interaction between the diet and weight gain, including that formula-fed infants gain more weight from 3 months of age compared to breastfed infants (two studies). They also discuss that it is not possible to reconcile variable findings of infant diet effects on the development of islet autoimmunity (seven studies), such as early introduction of cow's milk and cereals, by an overriding risk of weight gain (three studies). A limitation of the study is the outcome measure of islet autoimmunity rather than T1DM.

#### Reports

With regard to T1DM, EFSA (17) state that present available data on the risk of T1DM support the belief that gluten containing foods should be introduced not later than 6 months of age, preferably while still breastfeeding.

In a joint statement, COT/SACN (50) refute EFSA's conclusion on the introduction of gluten into the infant diet no later than 6 months of age with the aim of reducing the risk of subsequent development T1DM (17). COT/SACN (50) do not consider the evidence sufficient to support the precise statement about age, but considers the evidence strong for the protective effects of introduction of gluten while breastfeeding is continued.

With regard to T2DM, SACN (20) and WHO (21) both state that infants who are not breastfed are at greater risk of type 2 diabetes.

#### Conclusion

In conclusion, longer duration of breastfeeding may contribute to risk reduction in the development of T1DM according to a number of retrospective studies collected in a SLR (22). Longer breastfeeding seem to decrease the risk more than short-term breastfeeding. With regard to the reports by EFSA (19) and COT/SACN (50), introduction of gluten containing foods while still breastfeeding gives some protection against T1DM. Breastfeeding could also be considered a modifiable risk factor for the development of T2DM, and in the reports by SACN (20) and WHO (21) both state that infants who are not breastfed are at greater risk of T2DM.

In the present SLR, we therefore conclude that the evidence for any breastfeeding having a protective effect against T1DM and T2DM is probable (grade 2). The evidence for a stronger protective effect for longer duration of breastfeeding is still limited but suggestive (grade 3). It is unclear whether the positive effects seen for breastfeeding depend on the breast milk itself, on the avoidance of other foods given to infants, or on other factors that have been suggested in literature such as lower prevalence of infections in the breastfed child (see below).

### Acute otitis media, gastrointestinal infection, lower respiratory infection

Supplementary Table 5 shows studies with outcome acute otitis media (AOM), gastrointestinal infection (GI), and lower respiratory infection (LRI). In total, seven papers were chosen in the systematic review process to be evaluated for the evidence of an association between breastfeeding and infections. Of those, four were prospective cohorts (including birth cohort) and three were SLRs/MAs (of which two were graded A).

#### SLR/meta-analysis

Dujits et al. did a SLR (51), graded B due to no power calculation, including 21 studies from industrialized countries (defined by the World Bank as high income) (case-control, follow-up or randomized control trials); eight studies on GI and 16 studies on LRI with duration of follow-up 0–30 days and 0–24 months, respectively. Six out of eight studies suggested that breastfeeding had a protective effect on GI (the size of the effect varied according to duration and exclusiveness of breastfeeding), and 13 out of 16 studies concluded that breastfeeding had a protective effect against LRI. Five studies combined duration and exclusiveness of breastfeeding. All those studies observed a protective dose/duration-response effect on gastrointestinal or respiratory tract infections. These studies strongly suggest that breastfeeding protects infants against overall infections and gastrointestinal and respiratory tract infections in industrialized countries. The definitions of breastfeeding varied in the included studies and no description of the methodology used to assess dietary intake was given.

The A-graded SLR by Ip et al. (22), concluded that breastfeeding was associated with significant reduction in AOM, although only four of the five cohort studies included in the meta-analysis had clear definitions on feeding practices. For the final analysis infant feeding was dichotomized into two groups; exclusive breastfeeding and partial/mixed feeding vs. exclusive artificial feeding. Pooled adjusted OR of risk for AOM when comparing ever breastfed with never breastfed was 0.77 (95% CI: 0.64, 0.91). Comparing exclusively breastfed infants for 3 or 6 months compared with never breastfed gave a pooled adjusted OR 0.50 (95% CI: 0.36, 0.70). Results were conflicting for GI and the 16 studies (12 prospective cohort, two retrospective cohort, two case-control) included in a meta-analysis were graded B and suffered from various methodological flaws. For LRI a meta-analysis was done including seven cohort studies, the relative risk (RR) of hospitalization due to LRTI <1 year in those exclusively breastfed 4 months or more compared with formula-fed infants was 0.28 (95% CI: 0.14, 0.54).

Another A-graded SLR, Kramer and Kakuma (2), compared exclusive breastfeeding for 6 months vs. exclusive breastfeeding for 3–4 months with mixed breastfeeding including 22 studies from 11 developing and 11 developed countries (controlled clinical trials and observational studies). They reported that infants who continued exclusive breastfeeding for six months had a significantly reduced risk of one or more episodes of GI (RR 0.67 [95% CI: 0.46, 0.97]).

#### Prospective cohort studies

Dujits et al. also performed a cohort study in the Netherlands (52), graded B due to a lack of power calculation. They divided breastfeeding into five groups: 1) never (12.8%), 2) partial for <4 months, not thereafter (29.2%), 3) partial 4–6 months (28.8%), 4) exclusive for 4 months, partial thereafter (25.7%), and 5) exclusive for 6 months (1.4%). (Partial=breastmilk+formula and/or solids.) Compared with never breastfed, those exclusively breastfed 4 months+partially thereafter had lower risk of upper respiratory tract infection (URTI), LRTI, and GI until 6 months (adjusted OR [95% CI] 0.65 [0.51, 0.83], 0.50 [0.32, 0.79], and 0.41 [0.26, 0.64], respectively) and lower risk of LRTI between 7 and 12 months, adjusted OR 0.46 (95% CI: 0.31, 0.69). Partial breastfeeding, even for 6 months, did not result in significantly lower risks.

Fisk et al. (53), in a birth cohort study from Southampton, UK, compared gastrointestinal, respiratory, and ear infections during 0–6 months and 6–12 months between infants breastfed for seven different durations; never breastfed, <1 months, 1–3 months, 4+ months, 4–6 months, 7–11 months, 12+ months. Twenty-five percent of the infants were breastfed up to 6 months and 10% for 12 months or longer. Except for ear infections, an inverse dose-dependent relationship was found between breastfeeding duration and morbidity. Adjustment was done for several maternal and infant factors, including smoking in pregnancy and age at introduction of solid foods. Breastfeeding duration decreased the risk of diarrhea (adjusted RR, 95% CI) for breastfeeding >6 months vs. never breastfeeding, 0.43 (0.30, 0.61). The authors also identified that each month of additional breastfeeding decreased the risk of diarrhea. Adjusted RR per month increase in breastfeeding was 0.88 (0.83, 0.92) at 0–6 months, p for trend <0.001, and 0.97 (0.95, 0.99) at 6–12 months, p for trend=0.002. They found similar significant results for vomiting, wheezing, LRI, and general respiratory morbidity. There was a non-significant association between breastfeeding duration and prevalence of ear infection at 0–6 months and at 6–12 months.

Ladomenou et al. (54) studied all infections as one outcome. Prolonged exclusive breastfeeding was associated with fewer infectious episodes (r(s)=−0.07, *p*=0.019) and fewer admissions to hospital for infection (r(s)=−0.06, *p*=0.037) in the first year of life. Partial breastfeeding did not seem to have a protective effect. As for AOM, infants exclusively breastfed for 6 months presented with fewer infectious episodes than their partially breastfed or non-breastfed peers and this protective effect persisted after adjustment for potential confounders for AOM (OR 0.37 [95% CI: 0.13, 1.05]). A protective effect was also seen for acute respiratory infection (ARI) (OR 0.58 [95% CI: 0.36, 0.92]), and thrush (OR 0.14 [95% CI: 0.02, 1.02]).

Rebhan et al. (40) showed in a prospective cohort study that exclusive breastfeeding ≥6 months significantly reduced the risk for GI episodes during months 1–9 compared to those breastfed <4 months (includes never breastfed). Adjusted odds ratio (OR) was 0.60 and 95% CI 0.44–0.82. However, some important confounders were not included, the follow-up period was only 9 months, and no power calculations were done.

#### Conclusion

Based on the present SLR, we conclude that the evidence is convincing (grade 1) that breastfeeding protects infants in industrialized countries against overall infections, AOM, and gastrointestinal and respiratory tract infections. The magnitude of the effect varies depending on the specific outcome and the exclusiveness of breastfeeding. The definitions of breastfeeding varied in the included studies and the methodology used to assess breastfeeding was not always clear which is problematic. A protective dose/duration-response effect on gastrointestinal or respiratory tract infections was found in the SLRs of Dujits et al. (51) and Kramer (2), as well as in the prospective studies by Fisk et al. (53) and Ladomenou et al. (54).

### Cancer

Supplementary Table 6 shows studies with outcome childhood and adult cancers (details are provided in Appendix 3–5). In total three papers were found in the systematic review process; all were SLR/MAs. Of those, one was graded A (22) and two were graded C (55, 56). Neither of the C-graded SLR/MAs had used duplicate study selection and data extraction in the SLR, most of the included studies (>80%) relied on long-term recall of infant feeding, and moreover, in (55) only 8% examined breastfeeding exclusivity and control response rates were under 80% in over half. As there were so few studies with cancer as the outcome the two studies graded C are included below.

Ip et al. (22), graded A, was a systematic review of one SLR and one meta-analysis (only including case-control studies), both graded A by Ip et al. In addition, Ip et al. also conducted a new meta-analysis of three case-control studies. A total of 3,266 subjects with acute lymphocytic leukemia (ALL) were included in the three studies. There was an association between a history of breastfeeding of at least 6 months and a reduction in the risk of both ALL and acute myelogenous leukemia (AML). Breastfeeding ≤6 months vs. never breastfeeding: ALL OR 0.91 (95% CI: 0.83, 1.00), breastfeeding >6 months vs. never breastfeeding: ALL OR 0.80 (95% CI: 0.71, 0.91). Ip et al. conclude that there is association between a history of breastfeeding of at least 6 months duration and a reduction in the risk of both ALL and AML.

Martin et al. (55) did an SLR, graded C, on childhood cancers including 26 studies (mainly case-control) comparing ever or exclusive breastfeeding vs. never breastfed. Having been breastfed was associated with lower risks for acute lymphoblastic leukemia OR 0.81 (95% CI 0.84, 0.98), for Hodgkin's disease OR 0.76 (95% CI 0.60, 0.97) and for neuroblastoma OR 0.59 (95% CI 0.44, 0.78), with little between-study heterogeneity. However, even if causal, the authors state the public health importance of these associations may be small.

Martin et al. (56) also did an SLR, graded C, on adult cancers (breast, prostate, colorectal, gastric, smoking-related) including 14 studies (mainly case-control) also comparing ever or exclusive breastfeeding vs. never breastfed. Their conclusion was that ever having been breastfed was not associated with prostate, colorectal, gastric, smoking-related cancers, nor overall breast cancer risk RR 0.94 (95% CI: 0.85, 1.04). However, breastfed women had a reduced risk of premenopausal breast cancer RR 0.88 (95% CI: 0.79, 0.98) but not of postmenopausal breast cancer RR 1.00 (95% CI: 0.86, 1.16).

#### Conclusion

Based on the present SLR, we judge that there is limited but suggestive evidence (grade 3) for a risk reduction of breastfeeding against childhood leukemia and possibly other childhood cancers. The effect on childhood leukemia seems larger with longer breastfeeding duration (>6 months). However, as childhood cancers are relatively rare, the public health importance of these associations may be small. Research and evidence is too scarce and weak to judge associations between breastfeeding and cancers in adulthood.

### Atopic disease

Supplementary Table 7 includes 13 studies relating breastfeeding or introduction of solid foods to atopic disease (details are provided in Appendix 3–5). These include three SLRs/MAs and 10 prospective cohort studies. All but one SLR (22) were graded B.

#### Breastfeeding/exclusive breastfeeding

##### SLR/Meta-analysis

Ip et al. (22) made a meta-analysis of 18 prospective cohort studies with the outcome atopic disease. When comparing infants exclusively breastfed over 3 months vs. less than 3 months exclusively breastfed children with a family history of atopy the OR was 0.58 (95% CI: 0.41, 0.92). When separating those with short follow-up (<2 years) and those with longer ORs were 0.74 (95% CI: 0.61, 0.90) and 0.78 (95% CI: 0.62, 0.99), respectively. For those without a family history of atopy OR was 0.84 (95% CI: 0.59, 1.19).

Yang et al. (57) examined in a SLR/MA including 21 studies with 27 study populations, the association between exclusive breastfeeding for at least 3 months and the development of atopic dermatitis in childhood (1–7 years). There was no strong evidence of a protective effect of exclusive breastfeeding for at least 3 months against atopic dermatitis. As for the comparison group, exclusive breastfeeding <3 months or breastfeeding combined with formula feeding were defined as partial breastfeeding. Fifteen studies compared with partial breastfeeding and 6 studies compared with infant formula, cow's milk or soy milk. In summary, for the effect of exclusive breastfeeding on the risk of atopic dermatitis the OR was 0.89 (95% CI: 0.76, 1.04), and for study populations with atopic heredity a pooled OR was 0.78 (95% CI: 0.58, 1.05). The authors underline that due to substantial heterogeneity across studies, the results should be interpreted with caution.

##### Prospective cohort studies

Bergmann et al. (58) report on the association between total breastfeeding duration and the prevalence of eczema during the first 7 years in a German cohort. No consideration was given to other foods and infants breastfed <1 week is combined with those never breastfed. In total, 92% were breastfed at maternity ward, but 2% received glucose solution and 49% formula in addition (36% cow's milk formula and 13% hydrolyzed) which could have affected the results. Breastfeeding was carried out longer if at least one parent had eczema. Prevalence of eczema during first 7 years increased with each additional month of breastfeeding (OR 1.03 [95% CI: 1.00–1.06]), with a history of parental eczema (OR 2.06 [95% CI: 1.38, 3.08]), and if other atopic signs and symptoms appeared, especially specific sensitization (OR 1.53 [95% CI: 1.25, 1.88]), and asthma (OR 1.41 [95% CI: 1.07, 1.85]). Bergmann et al. conclude that parental eczema is the major risk factor, but longer duration of breastfeeding also increases the risk. Furthermore, although breastfeeding should be recommended for all infants, it does not prevent eczema in children with a genetic risk.

Elliott et al. (59) report on an analysis from a large prospective cohort study (Avon Longitudinal Study of Parents and Children [ALSPAC]) in England. They studied duration of breastfeeding and exclusive breastfeeding 2 and atopy (skin-prick test) at 7 years. Duration of any breastfeeding (never,B1 month, 1–3 months, 3–6 months, and 6– months) as well as exclusive breastfeeding (never breastfed, exclusively breastfed <4 months, exclusively breastfed ≥4 months) was compared with the outcomes. They found no consistent evidence for either a deleterious effect or a protective effect of breastfeeding on later risk of allergic disease, even when their mothers were asthmatic. Neither reverse causation nor low follow-up appears to have biased the results.

Giwercman et al. (60) studied duration of exclusive breastfeeding^3^ and eczema in the first 2 years of life in the Copenhagen Prospective Study on Asthma in Childhood (COPSAC) in a high-risk birth cohort (born to mothers with a history of asthma). As a definition of exclusive breastfeeding was not included, it is unclear what constitutes duration of breastfeeding. It was found that (exclusive) breastfeeding increased the risk of eczema after adjustment for demographics, filaggrin variants, parents’ eczema, and pets at home (n=306; RR 2.09 [95% CI: 1.15, 3.80]; *p*=0.016).

Three papers from the PROBIT-study focused on atopic disease as the outcome; allergy and asthma at 6.5 years evaluated through ISAAC questionnaire and skin-prick tests (61), allergy symptoms during the first 6.5 years evaluated through an ISAAC questionnaire (62), and atopic symptoms evaluated through skin-prick tests (37). The first paper reports that the experimental area had no reduction in risks of allergic symptoms and diagnoses or positive skin-prick tests (61). In fact, after exclusion of six sites (three experimental and three control) with suspiciously high rates of positive skin-prick tests, risks were significantly increased in the experimental group for four of the five antigens. The second paper reports that maternal postnatal smoking was associated with wheezing and hay fever symptoms, while the duration of exclusive breastfeeding was not protective against any of the studied outcomes (62). The risk factors for allergic symptoms were similar in children with positive skin-prick tests to those in the overall cohort. The third paper reports that no significant differences in atopic outcomes were found between the EBF3 and EBF6 groups (37).

Silvers et al. (63) studied the relationship between breastfeeding (exclusive and any) and doctor-diagnosed asthma, wheezing, inhaler use, and eczema at 15 months of age at 15 months of age in the New Zealand Asthma and Allergy cohort study.^4^ The median duration of exclusive breastfeeding was 1.4 months (interquartile range [IQR] 0–4) and of any breastfeeding was 9.0 months (IQR 4–13). Breastfeeding was not associated with eczema or atopy at 15 months.

#### Introduction of complementary foods

##### SLR/meta-analysis

Tarini et al. (64) conducted a SLR, including 13 studies (n=79–1,265) on early introduction of solid foods (defined as before age 4 months) and allergy. They concluded that early solid feeding may increase the risk of eczema. However, there were little data supporting an association between early solid feeding and other allergic conditions. The authors state that many of the reviewed studies lacked a rigorous design and so were susceptible to multiple biases. Five of nine studies found a positive association between early solid feeding and eczema, with persistence of the association for 10 years in one study. Another study found an association between early solid feeding and pollen allergy. No strong evidence was found to support the association between early solid feeding and the development of persistent food allergy, allergic rhinitis or animal dander allergy. In summary, the authors conclude that the evidence linking early solid feeding and allergic disease is inconsistent and conflicting.

##### Prospective cohort studies

Alm et al. (65) studied associations between patterns of food introduction and the risk of eczema. Food data were collected retrospectively at 6 and 12 months. Introduction of fish at <9 months of age decreased the risk, OR 0.76 (95% CI: 0.62, 0.94), *p*=0.009, but there was no effect of breastfeeding duration. Maternal eczema increased the risk, OR 1.54 (95% CI: 1.30, 1.84), as did having a sibling with eczema OR 1.87 (95% CI: 1.50, 2.33).

Snijders et al. (66) evaluated in a prospective birth cohort study (KOALA) from the Netherlands age of first introduction of cow's milk products and other food products and atopic manifestations in the first 2 years of life. Breastfeeding duration was included as a confounder. They found that more delay in both introduction of cow's milk products and other food products was associated with a higher risk for eczema at 2 years of age. No associations were found between introduction of cow's milk products and atopic dermatitis (AD); however, more delay in other food products was associated with a higher risk for AD. A delayed introduction of other food products was associated with an increased risk for atopic sensitization. Exclusion of infants with early symptoms of eczema (to avoid reverse causation) did not essentially change the results.

Zutavern et al. (67) reported from a prospective study in Germany (LISA birth cohort study) investigating timing of solid food introduction and skin and allergic symptoms at 6 years of age. They found that a delayed introduction of solids (between 4 and 6 months or past 6 months) was not associated with decreased odds for sensitization against food or inhalant allergens at 6 years of age. On the contrary, food sensitization was more frequent in children who were introduced to solids later. They concluded that they found no evidence supporting a delayed introduction of solids beyond 4 or 6 months for the prevention of allergic rhinitis and food or inhalant sensitization at the age of 6 years. For eczema, the results were conflicting and a protective effect of a delayed introduction of solids could not be excluded.

##### Reports

The American Association of Pediatrics (16) states that there is evidence that breastfeeding for at least 4 months, compared with feeding formula made with intact cow's milk protein, prevents or delays the occurrence of AD and cow's milk allergy in early childhood. There is little evidence that delaying the timing of the introduction of complementary foods beyond 4–6 months prevents the occurrence of atopic disease. At present, there are insufficient data to document a protective effect of any dietary intervention beyond 4–6 months for the development of atopic disease.

Swedish Pediatric Society (68) concludes that breastfeeding has not been proven to decrease the risk of atopy and allergies. Nor is there any evidence to indicate that it is preferable to avoid giving the baby allergenic foods or delay the introduction.

##### Conclusion

Based on the present SLR, we conclude that the existing scientific evidence is very limited and no conclusions (grade 4) can be drawn for any preventive effects of breastfeeding on atopic diseases in children. Of the two included SLR/MA studying the effect of exclusive breastfeeding >3 months on the risk for atopic disease, one found a protective effect (22), and the other found no significant effect regardless of heredity (57). The third SLR (64) looked at early introduction of solid food (<4 months) and concluded that early solid feeding may increase the risk for eczema, but that little data support an association between early solid feeding and other allergic conditions. The results from the prospective studies were similar. The prospective studies found no protective effect of exclusive breastfeeding on the development of atopic disease and the results from varying ages of introduction of solids were conflicting. Longitudinal studies in cohorts of newborn infants could help clarify the relationship of exclusively and/or duration of breastfeeding, as well as introduction of solid foods, and atopic diseases.

### Asthma

Supplementary Table 8 shows 14 studies relating breastfeeding or introduction of solid foods to asthma (details are provided in Appendix 3–5). These include three SLRs/MAs and 12 prospective cohort studies. All but one SLR (22) were graded B.

#### Breastfeeding/exclusive breastfeeding

##### SLR/meta-analysis

Ip et al. (22) did a meta-analysis of 15 prospective cohort studies (12 included in a previous meta-analysis graded A, and 3 newer studies all graded B). Ip et al. conclude that in children without a family history of asthma breastfeeding for more than 3 months was associated with reduced risk of asthma compared to not being breastfed (OR 0.73 [95% CI: 0.59, 0.92]). This association was also found in subjects <10 years of age with a family history of asthma.

Kramer and Kakuma (2) conducted a SLR (including controlled clinical trials and observational studies) on exclusive breastfeeding (6 months vs. exclusive 3–4 months with mixed breastfeeding) and wheezing or asthma. No significant reduction in the risk of asthma has been demonstrated. Risk of asthma at 5–6 years (pooled RR was 0.91 [95% CI: 0.61, 1.36]) and risk of wheezing in the exclusively breastfed (6 months) group was RR 0.79 (95% CI: 0.49, 1.28).

##### Prospective cohort studies

Elliott et al. (59) studied the association between breastfeeding and the outcomes wheeze at 3 and 7.5 years, asthma 7.5 years, and lung function at 8 years. Duration of any breastfeeding (never, B1 months, 1–3 months, 3–6 months and 6_months) as well as exclusive breastfeeding^5^ (never breastfeeding, exclusively breastfed <4 months, exclusively breastfed ≥4 weeks) was compared with the outcomes. Unfortunately, both wheeze and asthma was self-reported and there was no power calculation. No consistent evidence for either a deleterious effect or a protective effect of breastfeeding on later risk of allergic disease was found, even when the mothers were asthmatic. The authors state that neither reverse causation nor low follow-up appears to have materially biased the results.

Fredriksson et al. (69) studied breastfeeding duration and childhood asthma in a 6-year follow-up (children 7–14 years) population-based cohort study in Finland. Chronic respiratory symptoms (persistent wheezing, cough, phlegm) which could be indicators of future asthma were studied as secondary outcomes. A U-shaped relationship was found between breastfeeding duration and prevalence of asthma, wheezing, and phlegm. The lowest prevalence of asthma was found in children who were breastfed for 4–6 months and of chronic respiratory symptoms when the child was breastfed for 7–9 months. The adjusted OR for asthma was 1.03 (95% CI: 1.00, 1.05) per 1-month increase in breastfeeding duration for more than 6 months.

Giwercman et al. (60) reported from the Danish COPSAC study in a high-risk birth cohort on duration of exclusive breastfeeding^6^ and wheezy disorders during the first 2 years of life. The risk of wheezy disorders was reduced during the time the infant was (exclusively) breastfed. They found that (exclusive) breastfeeding reduced the risk of wheezy episodes in multivariate analysis adjusted for maternal smoking and age at start in day care (RR 0.67 [95% CI: 0.48, 0.96]; p–0.021) and of severe wheezy exacerbation (RR 0.16 [95% CI: 0.03, 1.01]; p–0.051).

Karmaus et al. (70) studied the triad of maternal prenatal smoking, any breastfeeding ≥3 months, and recurrent lower respiratory tract infection (RLRTI), and their association on childhood asthma 0–10 years. Of the three factors, RLRTI seemed to be the most important. Breastfeeding ≥3 months decreased the effects of both RLRTI and smoking on asthma.

Kramer et al. (61) reported from the PROBIT-study that there was no reduction in the risk of asthma at age 6.5 years when comparing the intervention with the control areas. This does not support the view that prolonged or exclusive breastfeeding has a protective effect on asthma or allergy. A second paper (62) reports that maternal postnatal smoking was associated with wheezing and hay fever symptoms, while the duration of exclusive breastfeeding was not protective against any of the studied outcomes. The risk factors for allergic symptoms were similar in children with positive skin-prick tests to those in the overall cohort.

Kull et al. (71) studied recurrent wheeze, asthma, lung function and, sensitization (specific IgE) at the ages 1, 2, 4, and 8 years in a birth cohort (BAMSE) in Sweden. Comparisons were made between exclusive vs. partial breast feeding, the durations of both were grouped into three categories (0 to <2, 2 to <4, and ≥4 months). The majority, 80% were exclusively breastfed during the first 4 months, mean duration 5.1 months (SD 2.5 months). At 8 years, exclusive breastfeeding for at least 4 months reduced risk for asthma (adjusted OR 0.63 [95% CI: 0.50, 0.78]) compared with breastfeeding <4 months, especially when combined with sensitization; the risk of allergic asthma was adjusted OR 0.59; 95% CI: 0.37, 0.93, while non-allergic asthma had an adjusted OR 1.18; 95% CI: 0.56, 2.48.

Midodzi et al. (72) studied several exposures (prenatal problems, cesarean delivery, low birth weight, breastfeeding, wheezing, allergy, infection, daycare) and the risk for asthma 0–5 years. Breastfeeding was defined as never, <3 months, and ≥3 months. Breastfeeding data were collected with a retrospective questionnaire at recruitment which occurred before 2 years. Breastfeeding was not a major interest in the study but was reported to decrease the incidence of asthma (breastfeeding >3 months HR: 0.82 [95% CI: 0.69, 0.97]).

Scholtens et al. (73) measured specific immunoglobulin E (IgE) to airborne allergen and bronchial responsiveness in 8-year-old children who participated in the PIAMA prospective birth cohort (Prevention and Incidence of Asthma and Mite Allergy). Breastfeeding >16 weeks vs. no breastfeeding was significantly associated with lower asthma prevalence from 3 to 8 years of age (OR=0.57 [95% CI: 0.41, 0.80]), this was also significant stratified in children of non-allergic fathers (OR=0.62 [95% CI: 0.40, 0.94]) and mothers OR 0.52 (95% CI: 0.34, 0.78) and children with allergic fathers OR 0.51 (95% CI: 0.30, 0.86). It did not reach significance for children with allergic mothers probably because of the low number of children in the group.

Silvers et al. (63) studied the relation between breastfeeding (exclusive and any) in relation to doctor-diagnosed atopy at 15 months of age in the New Zealand Asthma and Allergy cohort study. The median duration of exclusive breastfeeding was 1.4 months (interquartile range [IQR] 0–4) and of any breastfeeding was 9.0 months (IQR 4–13). Breastfeeding significantly reduced the risk of adverse respiratory outcomes at 15 months. Duration of exclusive breastfeeding was a stronger determinant of respiratory outcomes than the duration of any breastfeeding. After adjustment for confounders, each month of exclusive breastfeeding reduced risk of doctor-diagnosed asthma by 20% (OR 0.80 [95% CI: 0.71, 0.90]), wheezing by 12% (OR 0.88 [95% CI: 0.82, 0.94]) and inhaler use by 14% (OR 0.86 [95% CI: 0.78, 0.93]). Each month of any breastfeeding reduced the risk for these outcomes by 7–8%. Children with the lowest risk for asthma were exclusively breastfed for at least 3 months and continued breastfeeding reduced the risk even more.^7^

#### Introduction of complementary foods

##### SLR/meta-analysis

The SLR by Tarini et al. (64) on early introduction of solid foods (before age 4 months) concluded that no strong evidence was found to support the association between early solid feeding and the development of asthma and/or wheezing. The SLR included 13 studies (n=79–1,265). One case-control study found a positive association with asthma, while three cohort studies found no significant relationship with asthma by 4, 5, or 7 years. Furthermore, three cohort studies found no significant association with episodes of wheezing, while one found a positive association. In summary, the authors conclude that the evidence linking early solid feeding and allergic disease is inconsistent and conflicting.

##### Prospective cohort studies

Snijders et al. (66) reported from the KOALA study in the Netherlands, a prospective cohort study on age at first introduction of cow's milk products and other food products and atopic manifestations in the first 2 years of life. Breastfeeding duration was included as a confounder. A delayed introduction of other food products showed higher risk for recurrent wheeze. They found that longer breastfeeding duration (7–9 months) showed a reduced risk for recurrent wheeze, and the risk for recurrent wheeze for breastfeeding >9 months tended in the same direction.

Zutavern et al. (67) reported from a prospective study in Germany (LISA birth cohort study) studying feeding history at 6 months (solid food introduction) and skin and allergic symptoms at 6 years. They found that a delayed introduction of solids (between 4 and 6 months or past 6 months) was not associated with decreased odds for asthma at 6 years of age.

##### Reports

The American Association of Pediatrics (16) states that there is evidence that breastfeeding for at least 4 months, compared with feeding formula made with intact cow's milk protein, prevents or delays the occurrence of wheezing in early childhood.

Swedish Pediatric Society (68) concludes that breastfeeding gives some protection against infection-induced airway symptoms of asthma type.

##### Complementary search

Brew et al. (74) conducted a systematic review and meta-analysis including birth cohort, cross-sectional and case-control studies on breastfeeding and wheezing illness in children aged over 5 years, graded B (due to no statement about conflict of interest). Studies that measured any breastfeeding or exclusive breastfeeding for 3 or 4 months were included. Wheezing illnesses, including asthma, were identified based on symptoms, reported diagnosis or objective criteria. Meta-analysis of 23 studies that assessed any breastfeeding found that there was no overall association between breastfeeding and wheezing illness, however these studies were found to be very heterogeneous. Similarly, meta-analysis of 13 studies on exclusive breastfeeding for >3 or 4 months found no association between exclusive breastfeeding and wheezing illness. These studies were also found to be heterogeneous. Subgroup analyses found that any breastfeeding slightly lowers the odds of wheeze but slightly increases the odds of asthma defined by specific criteria. The authors point out that the difference in effects of breastfeeding according to the nature of the wheezing illness highlights the heterogeneous nature of the illness.

Brew et al. (75) graded B, analyzed data from two cohorts, CAPS in Australia and BAMSE in Sweden, which had reported different findings on the association between breastfeeding and asthma. The definitions for breastfeeding, asthma, and allergy were harmonized and only participants with a family history of asthma were included. Breastfeeding status, reported in infancy, was defined as fully breastfed for 3 months or longer and duration of any breastfeeding classified in months. They found that breastfeeding reduced the risk of asthma at 4, 5, and 8 years in children with a family history of asthma. Stronger effect was seen in the Swedish cohort.

Sonnenschein-van der Voort et al. (76) graded B, studied duration and exclusiveness of breastfeeding and asthma-related symptoms (including wheezing) in preschool children, as part of the prospective cohort study, the Generation R Study in the Netherlands. Compared with children breastfed for 6 months, those never breastfed had overall increased risks of asthma-related symptoms, and for wheezing the OR was 1.44 (95% CI: 1.24, 1.66). Similar associations were reported for exclusive breastfeeding, and non-exclusively breastfed for 4 months had increased risk of wheezing (OR 1.21 [95% CI: 1.09, 1.34]) compared with exclusively breastfed for 4 months. They concluded that shorter duration and non-exclusiveness of breastfeeding were associated with increased risk of asthma-related symptoms during the first 4 years of life, with the strongest effect estimates the first 2 years. Furthermore, that these associations seemed, at least partly, to be explained by infectious, but not by atopic mechanisms.

##### Conclusion

The studies included in the present SLR on the association between breastfeeding and asthma found contradictive results. One of the SLRs concluded that breastfeeding for 3 months or more diminished the risk of getting asthma (22), while the other found no significant effect when comparing exclusive breastfeeding for 3–4 months vs. 6 months (2). Similarly of 10 prospective studies, three found no effect (59, 61, 62), one found a U-shaped relation with the lowest prevalence of asthma with breastfeeding for 4–6 months (69), and the remaining six prospective studies found diminished wheeze or asthma risk associated with breastfeeding (60, 63, 70–73), of which one found a dose-response relationship (63). For the studies testing association between introduction of complementary foods and asthma (64, 66, 67), none found a significant effect. In conclusion, the present SLR found that the evidence linking breastfeeding or introduction of solid foods to asthma and wheeze is inconsistent, and the evidence is limited and no conclusions can be drawn (grade 4).

The complementary search found three papers which did not change our conclusion as they had differing results. An SLR (74) found in subgroup analyses that any breastfeeding slightly lowers the odds of wheeze but slightly increases the odds of asthma defined by specific criteria. However, in another study (75), data from two cohort studies (one Australian and one Swedish) were compared and found that breastfeeding reduced the risk of asthma at 4, 5, and 8 years in children with a family history of asthma. Stronger effect was seen in the Swedish cohort. The third study (76) found that shorter duration and non-exclusiveness of breastfeeding were associated with increased risk of asthma-related symptoms during the first 4 years of life, with the strongest effect estimates the first 2 years.

### IQ and neurological development

Supplementary Table 9 shows seven studies on breastfeeding and development in childhood and includes one SLR graded A (22), and six prospective cohort studies graded B (details are provided in Appendix 3–5). One additional prospective study was found through the complementary search.

#### SLR/meta-analysis

Ip et al. (22) did an SLR including one SLR rated A, two SLRs rated B, and eight new cohort studies (1A, 6B and 1C) and found little or no evidence for an association between breastfeeding in infancy and cognitive performance in childhood. Most studies did not differentiate between exclusive and partial breastfeeding, and their conclusions qualified with respect to the definitions used for cognitive performance.

#### Prospective cohort studies

Jedrychowski et al. (77) studied the association between exclusive breastfeeding of various durations and neurodevelopment over a 7-year follow-up. The authors differentiate between exclusive breastfeeding ≤3 months, 4–6 months, and >6 months. Complementary feeding is defined as never breastfed or mixed fed the first 3 months. The authors write that children breastfed exclusively for up to 3 months had intelligence quotients (IQs) that were on average 2.1 points higher compared to those mixed fed the first 3 months (95% CI: 0.24, 3.9); children breastfed for 4–6 months scored higher by 2.6 points (95% CI: 0.87, 4.27); and the benefit for children breastfed even longer (>6 months) increased by 3.8 points (95% CI: 2.11, 5.45).^8^

In two papers from the PROBIT-study, Kramer et al. focus on neurological development (37, 78). First comparisons of IQ at 6.5 years between the intervention area and the control area were performed, n=13,889 (78). They conclude that the experimental area had higher means on all of the Wechsler Abbreviated Scales of Intelligence measures, with cluster-adjusted mean differences (95% CI) of 7.5 (0.8, 14.3) for verbal IQ; 2.9 (−3.3, 9.1) for performance IQ; and 5.9 (−1.0, 12.8) for full-scale IQ. Academic ratings by teachers were significantly higher in the experimental group for both reading and writing. In the second paper (37), they compared 2,951 out of 3,483 total participants followed during the first year. They found no significant differences between the EBF3 and EBF6 groups on Wechsler Abbreviated Scales of Intelligence measures, or teacher ratings on those that had started school.

Oken et al. (79) studied developmental milestones at 18 months among 25,446 children. Breastfeeding exposure (any) was divided into <1 month, 2–3 months, 4–6 months, 7–9 months, and >10 months. Children breastfed 2–3, 4–6, and >6 months all showed higher motor developmental milestones and total developmental milestones in comparison to those breastfed <1 month. Breastfed >6 months also showed higher social or cognitive developmental milestones in comparison to breastfed <1 month. Unfortunately, the study did not include exclusive breastfeeding, there were no power calculations, and study power and sample size was not considered although the study included very many participants.

Whitehouse et al. (80) showed that the positive associations of breastfeeding on language ability found at 5 years of age in the Australian Raine Study, were still present at the age of 10 years. Predominately breastfeeding was presumed to occur up to introduction of milk other than breast milk, and the definition did not preclude solids. A dose–response relationship was found between the duration of predominately breastfeeding and language ability at 10 years, adjusted for several potential covariates, including maternal education. Those predominately breastfed for <4 months had higher language scores than those never breastfed (regression coefficient [β]=2.71), while the effect was stronger for predominately breastfed for 4–6 months (β=3.83) and stronger still for predominately breastfed for >6 months (β=4.04). The magnitude of the dose-response association between predominant breastfeeding and higher language scores at 10 years was comparable to the effect found at 5 years.

Zhou et al. (81) studied associations between breastfeeding duration and IQ at 4 years of age in a prospective cohort study in Australia. The participants were children from a trial that investigated iron-supplementation in pregnancy. Duration of breastfeeding was defined as duration of any degree of breastfeeding (exclusive or partial). Children who were breastfed for at least 6 months had higher IQ than those who were breastfed for shorter duration. However, when adjusted for socioeconomic characteristics, the association between breastfeeding duration and IQ of the children was no longer significant. They found that the strongest predictor of IQ at 4 years was the quality of the home environment.

#### Reports

A report from WHO (21) concludes that subjects who were breastfed showed higher performance in intelligence tests. All effects were statistically significant, but for some outcomes their magnitude was relatively modest.

#### Complementary search

Oddy et al. (82), graded B, examined the relationship between breastfeeding for 4 months or longer and child development at age 1, 2, and 3 years in the Raine study. Infant feeding data were collected at each age. Breastfeeding (any breastfeeding) for 4 months or longer compared with breastfeeding for less than 4 months was associated with small but positive increases in psychomotor development scores, like fine motor skills, adaptability, and communication scores, from age 1–3 years. The authors concluded that although the effect sizes were small, breastfeeding for 4 months or longer were associated with improved developmental outcomes for children aged 1–3 years after adjustment for multiple confounders.

#### Conclusion

In their SLR, Ip et al. (22) conclude that they saw little or no evidence for a positive association between breastfeeding and later cognitive performance of the child. However, of the six later prospective cohort studies, four found positive associations between breastfeeding and increased IQ or developmental scores (77, 79–81). Two of these even found a stepwise increase with longer duration of breastfeeding with highest IQ points or developmental scores with breastfeeding longer than 6 months (77, 80). The positive results from the PROBIT-study when comparing the intervention and control areas (78) should also be seen as quite strong evidence for positive associations, while the non-results in their later paper (37) probably can be explained by the fact that they compared children exclusively breastfed for 3 or 6 months where differences are likely to be smaller than in the other studies where comparisons were made with children who were never breastfed, mixed fed or breastfed <1 month. The last study (81) found a positive association which was attenuated and no longer significant after adjustment for socioeconomic characteristics.

Based on the present SLR, we conclude that there is probable evidence (grade 2) that breastfeeding is beneficial for IQ and developmental scores of children, with increasing benefit with increasing duration. One study was found by the complementary search and it supported the conclusion that breastfeeding is beneficial for neurodevelopment (82).

### Celiac disease

Supplementary Table 10 shows one SLR graded A with outcome celiac disease and the association with breastfeeding; Akobeng et al. (83). This was a systematic review and meta-analysis of six observational case-control studies of various size and age. The SLR included a total of 1,131 cases and 3,493 controls (varying between 7 and 491, and between 73 and 1,949, respectively, in the different studies). The ages varied between 2 and 15 years. The SLR only included studies based on histologically confirmed celiac disease, but the primary studies compared different durations of breastfeeding and exact timing of introduction and amount of gluten consumed was not given. All included studies found a negative association between breastfeeding and celiac disease. The risk was especially reduced if the child was still breastfed when gluten was introduced (pooled OR 0.48 [95% CI: 0.40, 0.59]). However, the authors make the observation that it is not clear whether breastfeeding only delays the onset of celiac disease or if it provides permanent protection.

#### Reports

With regard to celiac disease, EFSA (17) state that present available data support the belief that gluten containing foods should be introduced not later than 6 months of age, preferably while still breastfeeding.

ESPGHAN (19) consider it prudent to introduce gluten in small amount while the infant is still breastfed and to avoid both early (<4 months) and late (>7 months) introduction of gluten.

In a joint statement, COT/SACN (50) state that they considers the evidence strong for the protective effects of introduction of gluten while breastfeeding is continued, but do not consider the evidence sufficient to support the precise statement about age at introduction of gluten (except that introduction should not occur before 3 months).

#### Conclusion

Based on the present SLR, we judge it to be probable evidence (grade 2) for breastfeeding as a protective factor for celiac disease, if gluten is introduced in small amounts while still breastfeeding, although it is unclear whether the protection only delays the onset of celiac disease or if it provides permanent protection. This conclusion is in line with the reports from ESPGHAN (19), EFSA (17), and COT/SACN (50). ESPGHAN (19) also considers it prudent to introduce gluten in small amounts. However, the evidence is limited and insufficient (grade 4) to conclude which age is best for introduction of gluten.

### Inflammatory bowel disease

Supplementary Table 11 shows one SLR graded A with outcome inflammatory bowel disease (IBD) and the association with breastfeeding; Klement et al. (84). This SLR included 17 studies including 15 retrospective case-control studies; 11 investigated both ulcerative colitis and Crohn's disease, three investigated ulcerative colitis alone, and three investigated Crohn's disease alone. Together a total of 2,577 patients with ulcerative colitis and 3,551 control subjects and 3,190 patients with Crohn's disease and 4,026 control subjects were studied. Approximately one quarter of the studies included adults only (>18 year), one quarter children (0–18 years), quarter a mix, and a quarter was unknown. Seven of the included studies were graded A. Breastfeeding had a statistically significant protective role against ulcerative colitis and an even greater role against Crohn's disease. Pooled OR for Crohn's disease was 0.45 (95% CI: 0.26, 0.79) and for ulcerative colitis 0.56 (95% CI: 0.38, 0.81) if only the studies of good quality are included. When all studies were included in the pooled estimate, the random-effects model OR was 0.77 (95% CI: 0.61, 0.96) for ulcerative colitis and 0.67 (95% CI: 0.52, 0.86) for Crohn's disease.

#### Conclusion

Based on the present SLR, we judge there to be probable evidence (grade 2) that breastfeeding provides protection against IBD. The conclusion is based on the SLR above which included 17 studies of which seven was graded A. However, there is insufficient evidence to give exact estimates of the risk reduction. Well-performed prospective studies with reliable, well-defined breastfeeding data are needed.

## Discussion

The overall aim of this systematic review was to evaluate recent scientific data on the short- and long-term health effects of breastfeeding (duration of any and exclusive breastfeeding) on the child and introduction of foods other than breast milk in order to assess the validity of the current Nordic recommendations, NNR4 (12). A second aim was to provide a background for the planned update on the chapter on breastfeeding.

Five research questions were developed involving 12 different outcomes and studies related to these have been presented in this review. A summary of the grading of the evidence for the various outcomes is presented in Supplementary Table 12. It should be emphasized that the grading of evidence is only based on studies from year 2000 and onwards, searched for in June 2011, although many earlier studies are part of the included SLRs/MAs. We also excluded all papers on specific constituents of breast milk, i.e. nutrients, biologically active substances, and contaminants. A complementary search was performed in January 2012, and the abstracts were evaluated for full paper reading. Complementary papers were used to evaluate the conclusion of the SLR, as supporting or not.

### Summary of results

We found the evidence convincing (grade 1) of a protective dose/duration effect of breastfeeding against overweight and obesity in childhood and adolescence, as well as against overall infections, AOM, and gastrointestinal and respiratory tract infections.

The evidence was probable (grade 2) that exclusive breastfeeding for longer than 4 months is associated with slower weight gain during the second half of the first year, compared with shorter duration, but no negative health effects are reported, rather that the slower growth in infancy helps reducing risk of later overweight or obesity. It is also of probable evidence that breastfeeding provides a small but significant reductive effect on blood pressure and on blood cholesterol later in life. Whether this has any effect on the risk of CVD is, however, unclear. The evidence is probable for beneficial effects of breastfeeding on IQ and developmental scores of children. Breastfeeding was also found to be a protective factor against IBD and celiac disease; the latter if gluten is introduced while still breastfeeding. There was also of probable evidence that any breastfeeding is protective against T1DM and T2DM, but the evidence for a larger protective effect of a longer duration of breastfeeding is still limited even though suggestive (grade 3).

There is also limited but suggestive evidence (grade 3) for a risk reduction of breastfeeding against childhood leukemia and possibly other childhood cancers. The effect seems larger on childhood leukemia with longer breastfeeding duration (>6 months). However, as childhood cancers are relatively rare, the public health importance of these associations may be small. Research and evidence is too scarce and weak to judge associations between breastfeeding and cancers in adulthood (grade 4).

Longitudinal studies in cohorts of newborn infants could help clarify the relationship of exclusive breastfeeding and/or duration of any breastfeeding, as well as introduction of solid foods, on the risk of atopic diseases, asthma, wheezing, and eczema. The evidence was insufficient, and no conclusion could be drawn (grade 4) for these associations nor about whether any specific age is more advantageous for introduction of gluten to protect against celiac disease. Other associations explored were also inconclusive.

### Discussion about specific outcomes

#### Growth, overweight, and obesity

The physiological explanations for the protective effect of breast milk from development of overweight are not totally clear and might partly rely on other foods introduced as well. Selective reporting and/or publication bias cannot be totally excluded in this area. Well-performed prospective studies with longer duration of breastfeeding as well as follow-up data are needed to evaluate breastfeeding association with growth and/or body composition later in life. Nordic collaboration with data from prospective longitudinal infant cohorts is urgent as they likely provide the best possibilities for improved studies on longer duration of both exclusive and any breastfeeding relevant to the Nordic populations.

Kramer et al. (38) found that smaller size (especially weight for age) was strongly associated with increased risks of subsequent weaning and of discontinuing exclusive breastfeeding (adjusted OR varied between 1.2 and 1.6), especially between 2 and 6 months. This is problematic as this may interfere with the possibility of drawing appropriate conclusions, and specifically it can be questioned if the longer duration of exclusive breastfeeding in e.g. (37) really is the cause of larger size at 6.5 years or a confounder.

The first results from a RCT-study situated in a developed country (Iceland) have recently been published (85). Infants taking part in the study were exclusively breastfed and randomized to introduce complementary foods at 4 or 6 months while continuing to breastfeed. In total 119 infants were recruited and 100 (50/group) completed the protocol. This first paper includes results from measurements at 6 months on intake of breast milk and other foods, anthropometry, and body composition. The study showed similar energy intake, growth and body composition whether exclusive breastfeeding continued for 4 or 6 months.

Previous growth charts were based on infants fed primarily infant formula which made them unsuitable for infants fed according to the WHO recommendations of exclusive breastfeeding for 6 months (86). Based on large global samples of prospectively followed, breastfed infants the new WHO child growth standards (45) bring several advantages compared with the old growth references. The opinion has been expressed that the new WHO standards will both take the pressure of breastfeeding women to give their babies formula or other food too early, and decrease the risk of overfeeding as the new chart gives a more realistic view of children growth (87).

#### Blood pressure, serum cholesterol, and CVD

The significant lower blood pressure (just over 1 mm Hg systolic) seen in adults breastfed in infancy compared with those not breastfed can be of less value to the individual but important in a public health perspective. One theoretical calculation from the mid-90s state that a decrease of 2 mm Hg of the mean blood pressure in the US population would result in 17% fewer people with high blood pressure, 6% fewer people with CVD, and 15% fewer people with stroke/transient ischemic attacks (88). Another study calculated that each increment of 20 mm Hg of systolic blood pressure and 10 mm Hg of diastolic blood pressure doubles the risk for CVD (89). How large effect a relatively small decrease in systolic blood pressure, such as that associated with breastfeeding, would have on CVD risk is unclear. No effects on blood pressure in childhood were seen.

Breastfeeding was also found to result in a small decrease of serum cholesterol in adults, while the effect in childhood was unclear. In an SLR included in Ip et al. (22), breastfed infants had higher cholesterol levels in 25 out of 26 studies but as that SLR was graded C no conclusions could be drawn about the evidence level. Whether the increased level in adults would have any effect on CVD risk is unclear. In the present SLR, CVD mortality was not included as an outcome. However, it was studied in the SLR by Ip et al. (22), comprising an SLR/MA (graded B) of four historical cohorts. Their conclusion was that the data reviewed provided no evidence that breastfeeding was related to all-cause or CVD mortality, but that more studies were needed due to possible sources of bias and limitations in the four included studies.

#### Diabetes mellitus, type 1 and 2 (T1DM and T2DM)

The evidence for any breastfeeding having a protective effect against T1DM and T2DM is probable (grade 2). The evidence for a stronger protective effect with longer duration of breastfeeding is still limited but suggestive (grade 3). However, it is unclear whether the positive effects seen for breastfeeding depends on the breast milk itself, on the avoidance of other foods given to infants, or on other factors such as decreased number of infections for the breastfed child. More studies are needed to clarify this.

#### Infections

The evidence that breastfeeding protects also infants in industrialized countries against overall infections, AOM, and gastrointestinal and respiratory tract infections is convincing (grade 1). The magnitude of the effect varies depending on the specific outcome and the exclusiveness of breastfeeding. A protective dose/duration–response effect on gastrointestinal or respiratory tract infections has been seen in several well-conducted studies.

#### Cancer

Two of the three SLRs found were graded C due to long-term recall of breastfeeding data and no duplicate study and data extraction. Based on three SLRs there we judge the evidence to be limited but suggestive (grade 3) for a risk reduction of breastfeeding against childhood leukemia and possibly other childhood cancers. The effect on childhood leukemia seems larger with longer breastfeeding duration (>6 months). However, as childhood cancers are relatively rare, the public health importance of these associations may be small. Research and evidence is too scarce and weak to judge associations between breastfeeding and cancers in adulthood.

#### Atopy and asthma

The evidence is insufficient and no conclusion could be drawn (grade 4) for the relationship of exclusive breastfeeding and/or duration of any breastfeeding, as well as introduction of solid foods, on the risk of atopic diseases, asthma, wheezing, and eczema. Previous advice on allergy prevention has included delayed introduction of other foods, elimination of various foods as well as active prevention by adding specific components either to the pregnant/lactating woman or infant diet. These recommendations have also changed over time.

It is well established that certain foods are more allergenic than others (i.e. milk, eggs, fish, nuts, and shellfish), but during the last decade there has been debate about whether and to what degree breastfeeding protects against atopic disease and asthma. The Swedish Pediatric Society (68) conclude that breastfeeding gives some protection against infection-induced airway symptoms of asthma type, but state that breastfeeding has not been proven to decrease the risk of atopy and allergies. Furthermore they state that there is no scientific evidence that the risk of allergic disease in the infant is reduced if the mother avoids certain foods during pregnancy or breastfeeding. There is also no evidence to indicate that it is preferable to avoid giving the baby allergenic foods or to delay the introduction. Any positive effect of giving different dietary supplements (n-3 fatty acids, pre-and probiotics, or vitamins) remains to be shown. The same applies to any advantage of the introduction of certain food allergens before 6 months of age and/or during breastfeeding. Introduction of other foods including how timing, food choices, and amounts may affect the child's health in the short- and long-term is thus a relatively unknown area, but several interesting studies have been initiated.

Silvers et al. (63) found no association between breastfeeding with eczema or atopy at 15 months, but a significant dose-dependent effect on asthma, wheezing, and inhaler use. They did not take other foods into account.

Snijders et al. (66) reported from the KOALA study in the Netherlands, a prospective cohort study on age at first introduction of cow's milk products and other food products and atopic manifestations in the first 2 years of life. A delayed introduction of other food products showed higher risk for recurrent wheeze. They found that longer breastfeeding duration (7–9 months) showed a reduced risk for recurrent wheeze, and the risk for recurrent wheeze for breastfeeding >9 months tended in the same direction. A question is if this, i.e. a higher risk related to delayed introduction of other foods and at the same time a protective effect of longer breastfeeding duration, should be interpreted as a protective effect of introduction of solids while breastfeeding continues, similar to the effect seen for celiac disease. There are some ongoing studies that might shed light on this in the future.

Even though the evidence was deemed to be inconclusive and only grade 4 partly due to contradictive results, it is however not far from grade 3. Future studies should make sure to include all possible confounders, use good definitions of feeding and doctors giving diagnosis; all important factors to make it possible to compare studies.

#### IQ and neurological development

Not all studies find a beneficial effect of breastfeeding on IQ and neurological development but no studies have found detrimental effects or that formula feeding should be advantageous in comparison. For instance, an earlier meta-analysis by Anderson et al. (90) found a positive effect while the SLR by Ip et al. (22) (where Anderson et al. was included) concluded that they saw little or no significant effects. However, as several strong cohort studies published after the SLR by Ip et al. (22) show positive effects of breastfeeding, and the few studies showing no or non-significant effects can be explained, we conclude that evidence is probable (grade 2) that breastfeeding is beneficial for IQ and developmental scores of children, with increasing benefit with increasing duration.

#### Celiac disease

With regard to celiac disease we judge the evidence to be probable (grade 2) for breastfeeding as a protective factor for celiac disease, but the evidence is insufficient (grade 4) to conclude which age is best for introduction of gluten. The most prudent way to introduce gluten is to introduce it in small amounts while still breastfeeding. However, it is unclear whether the protection only delays the onset or if it provides permanent protection. A large screening study has been conducted in Sweden (‘Exploring The Iceberg of Celiacs in Sweden’ (ETICS), www.etics.se) to shed light on this through screening at 12 years of age of two birth cohorts born in 1993, during the height of the Swedish ‘epidemic’ of clinical celiac disease in children <2 years of age, and in 1997 after it had ended. Recent data from this study show that children born in 1997, when a larger proportion were introduced to gluten in small amounts while still breastfeeding, had a significantly lower risk of having celiac disease compared with those born in 1993 (prevalence ratio: 0.75 [95% CI: 0.60, 0.93]; *p*=0.01) (91). This proves that the recommendation regarding introduction of gluten is favorable at least until 12 years of age.

#### Inflammatory bowel disease

We judge there to be probable evidence (grade 2) that breastfeeding provides protection against IBD, but the evidence is insufficient (grade 4) to give exact estimates of the risk reduction. Well-performed prospective studies with reliable, well-defined breastfeeding data are needed to enable such estimates.

### Difficulties with interpreting breastfeeding research

It is difficult to ascertain whether the positive effects seen for breastfeeding depend on the breast milk itself and its unique composition, on the avoidance of certain other foods given to the infant, the action of breastfeeding or on other associated factors. However, the nutrients and biologically active substances in breast milk are numerous, including vitamins, minerals, fatty acids, and various immune factors and many of these have proven positive effects on health.

Methodological problems with breastfeeding studies include the following: long recall, poor definition of exclusive breastfeeding, comparisons only between ever-never breastfed, and no details about what infants eat instead of or in addition to breast milk. In spite of the quality criteria used in the present SLR, the definitions of breastfeeding varied in the included studies and the methodology used to assess breastfeeding was not always clear which is problematic. As expected the strongest evidence was found when comparing exclusive breastfeeding with never breastfed.

In addition to the difficulties mentioned above there are many other confounding factors when studying the associations between infant feeding and health outcomes. Since breastfeeding is influenced by many different health-related factors, e.g. education, it can be difficult to safely conclude that it is feeding itself that explain positive health outcomes. The interpretation of epidemiological studies with regard to infant feeding is further complicated because the health outcome is not only influenced by whether the child is breastfed or not, what children are given instead of breast milk, and the exposure this gives, but also, for example, the facilitating effects breastfeeding have on mother–infant bonding. The latter may for instance have effects on development.

Well-performed prospective studies with well-defined infant feeding methods are needed to evaluate breastfeeding association with several of the outcomes. Nordic collaboration with data from prospective longitudinal infant cohorts would be valuable as there are probably good possibilities of methodologically strong studies on longer duration of both exclusive and any breastfeeding.

### The importance of considering the aim of a study when comparing results

Exclusive breastfeeding for about 6 months is recommended by most official bodies, e.g. AAP (15, 16), EFSA (17), ESPGHAN (18, 19), SACN (20), and WHO (1–3, 92). At the same time, EFSA (17) and ESPGHAN (19) talks about introducing solid food between 4 and 6 months of age. This may seem contradictory, but depends on different starting points for the scientific reviews behind the recommendations. While the starting point for WHO's review (21) was to answer the question of whether exclusive breastfeeding for 6 months is safe, EFSA (17) wanted to answer the question whether there are any disadvantages with starting to give complementary foods in addition to breastfeeding in the age range of 4–6 months in Europe. Knowledge of this difference and that the results do not conflict with each other is important. If a mother continues to breastfeed after 6 months, there is no scientific evidence that the introduction of complementary foods between 4 and 6 months would result in any health drawbacks for her child, but there is also no evidence that it would bring any health benefits.

A recent Cochrane review update by Kramer and Kakuma (92) assessed the effects on child health, growth, and development comparing exclusive breastfeeding for 6 months vs. exclusive breastfeeding for 3–4 months followed by mixed breastfeeding through 6 months. They share some, but not all, of the conclusions in the present SLR on health effects of exclusive breastfeeding. With regard to GI and allergic disease they draw the same conclusions, i.e. a protective effect for infection and no long-term effects for allergy. However, they also state that exclusive breastfeeding for 6 months does not seem to confer any long-term positive effects with regard to obesity and cognitive ability compared to exclusive breastfeeding for 3–4 months. The reason for the discrepancy is most likely that the aim of the new Cochrane-update by Kramer and Kakuma (92) differs from the aim of the present SLR. They only included studies comparing infants who were exclusively breastfed for at least 6 months followed by mixed breastfeeding, with infants introduced to liquid or solid foods between 3 and 6 months of age followed by mixed breastfeeding until 6 months or beyond. Thus their compared groups differed less than the groups compared in most of the studies included in the present SLR.

### Concerns about vitamin D and iron

Breastfeeding has been questioned about whether it gives enough vitamin D and iron to the breastfed infant. With regard to vitamin D it has long been known that infants and young children living in northern latitudes need vitamin D supplements (especially the exclusively breastfed infants) at least for some years. The experts on vitamin D in the NNR5-project has focused their SLR on what levels of supplementation should be recommended to different age groups and thus, this is not part of the present SLR.

There has also been concern that some infants will experience negative effects on iron status if breastfed exclusively for 6 months, and Kramer and Kakuma (92) note that a reduced level of iron has been observed in developing-country settings. The recent RCT-project from Iceland mentioned above has also a paper on ferritin levels accepted for publication (93). They report that at 6 months of age, the ferritin levels were lower in the group exclusively breastfed for 6 months compared with infants exclusively breastfed for 4 months and then receiving small amounts of complementary food in addition to breast milk until 6 months, but there was no indication or evidence that the difference was of biological or clinical importance.

## Conclusions

Convincing and probable evidence was found for benefits of breastfeeding on several outcomes. It is concluded that the recommendation about exclusive breastfeeding until 6 months of age from NNR2004 can stand unchanged as well as the recommendation about breast milk as part of the diet throughout the first year, and that partial breastfeeding can be continued as long as it suits mother and child. Considering the relatively low proportion of infants in the Nordic countries following this recommendation, strategies that protect, support and promote exclusive breastfeeding for around the first 6 months of an infant's life should be enhanced, and should recognize the benefits for long-term health.

## Footnotes

1 Solids not included in definition.

2 No definition of exclusive breastfeeding.

3 No definition of exclusive breastfeeding.

4 Other food was not taken into account.

5 No definition of exclusive breastfeeding.

6 No definition of exclusive breastfeeding.

7 Other food was not taken into account.

8 Exclusive breastfeeding is defined in accordance with WHO, but it is unclear whether it is actually used in practice, as 25% of infants are reported to be exclusively breastfed >6 months, which seem unlikely.
